# Novel Targets of Sulforaphane in Primary Cardiomyocytes Identified by Proteomic Analysis

**DOI:** 10.1371/journal.pone.0083283

**Published:** 2013-12-11

**Authors:** Cristina Angeloni, Silvia Turroni, Laura Bianchi, Daniele Fabbri, Elisa Motori, Marco Malaguti, Emanuela Leoncini, Tullia Maraldi, Luca Bini, Patrizia Brigidi, Silvana Hrelia

**Affiliations:** 1 Department for Life Quality Studies, Alma Mater Studiorum - University of Bologna, Bologna, Italy; 2 Department of Pharmacy and Biotechnology, Alma Mater Studiorum - University of Bologna, Bologna, Italy; 3 Functional Proteomics Laboratory, Department of Life Sciences, University of Siena, Siena, Italy; 4 Department of Surgical, Medical, Dental and Morphological Sciences with interest in Transplant, Oncology and Regenerative Medicine, University of Modena and Reggio Emilia, Modena, Italy; University of Virginia, United States of America

## Abstract

Cardiovascular diseases represent the main cause of mortality in the industrialized world and the identification of effective preventive strategies is of fundamental importance. Sulforaphane, an isothiocyanate from cruciferous vegetables, has been shown to up-regulate phase II enzymes in cardiomyocytes and counteract oxidative stress-induced apoptosis. Aim of the present study was the identification and characterization of novel sulforaphane targets in cardiomyocytes applying a proteomic approach. Two-dimensional gel electrophoresis and mass spectrometry were used to generate protein profiles of primary neonatal rat cardiomyocytes treated and untreated with 5 µM sulforaphane for 1-48 h. According to image analysis, 64 protein spots were found as differentially expressed and their functional correlations were investigated using the MetaCore program. We mainly focused on 3 proteins: macrophage migration inhibitory factor (MIF), CLP36 or Elfin, and glyoxalase 1, due to their possible involvement in cardioprotection. Validation of the time-dependent differential expression of these proteins was performed by western blotting. In particular, to gain insight into the cardioprotective role of the modulation of glyoxalase 1 by sulforaphane, further experiments were performed using methylglyoxal to mimic glycative stress. Sulforaphane was able to counteract methylglyoxal-induced apoptosis, ROS production, and glycative stress, likely through glyoxalase 1 up-regulation. In this study, we reported for the first time new molecular targets of sulforaphane, such as MIF, CLP36 and glyoxalase 1. In particular, we gave new insights into the anti-glycative role of sulforaphane in cardiomyocytes, confirming its pleiotropic behavior in counteracting cardiovascular diseases.

## Introduction

Coronary heart disease represents the main cause of mortality in the industrialized world, being responsible of over 40% of all deaths in Western Europe and United States [1]. Many studies have shown a critical role for oxidative stress in various forms of cardiovascular diseases, including myocardial ischemia-reperfusion (I/R) injury, congestive heart failure, atherosclerosis, and chemical-induced cardiotoxicity [2,3]. An emerging strategy to counteract oxidative cardiac damage is the up-regulation of endogenous antioxidants and phase II enzymes in cardiac cells by synthetic and naturally occurring agents [4,5]. Among them, sulforaphane (SF) (1-isothiocyanate-(4R)-(methylsulfinyl)butane) is one of the most promising diet-derived indirect antioxidant agents [6,7]. SF is produced by the breakdown of glucoraphanin, a glucosinolate abundantly present in some Cruciferous vegetables, especially broccoli [8]. It has been reported that broccoli protects hearts against I/R injury through the redox cycling of the thioredoxin superfamily [9]. A study conducted in 12 healthy subjects has shown that only one-week intake of broccoli sprouts improved cholesterol metabolism and decreased oxidative stress markers [10]. Moreover, broccoli consumption was strongly associated with reduced risk of cardiovascular heart disease death in postmenopausal women [11]. We have recently demonstrated that SF protects cardiomyocytes against apoptosis induced by oxidative stress [12]. In particular, SF cardioprotection was related to the up-regulation of a panel of key cellular antioxidants and phase II enzymes, including glutathione, glutathione reductase, glutathione-S-transferase, thioredoxin reductase, and NAD(P)H:quinone oxidoreductase 1, but other mechanisms could be involved in SF cardioprotective effects.

The present study aimed to identify and characterize novel targets of SF using a proteomic approach based on two-dimensional gel electrophoresis (2-DE) and mass spectrometry (MS). We focused our attention on 3 proteins: macrophage migration inhibitory factor (MIF), CLP36, and lactoylglutathione lyase, also known as glyoxalase 1 (GLO1), due to their possible involvement in cardioprotection and, in particular, on the cardioprotective role of GLO1 up-regulation by SF. In normal physiological condition GLO1 controls methylglyoxal (MG) homeostasis [13]. MG is the most investigated advanced glycation end product (AGE) precursor. AGEs are a heterogeneous group of molecules that are generated through non-enzymatic glycation and oxidation of proteins, lipids and nucleic acids [14]. Studies have shown that GLO1 overexpression has beneficial effects, such as prevention of myocardial cell death during reperfusion and delay of aging and senescence [15,16].

Our results suggested that SF cardioprotection is a complex mechanism involving not only the induction of phase II enzymes [12,17], but also unexpected proteins with anti-apoptotic role [18], acting as adapters between kinases and cytoskeleton [19], and counteracting AGE production. 

## Materials and Methods

### Materials

PhosSTOP was purchased from Roche Diagnostics (Mannheim, Germany). D,L-Sulforaphane (SF) was obtained from LKT Laboratories (Minneapolis, MN, USA). Anti-CLP36, anti-glyoxalase I (GLO1) and anti-macrophage migration inhibitory factor (MIF) were purchased from Santa Cruz Biotechnology (Santa Cruz, CA, USA). Anti-caspase-3, anti-rabbit and anti-mouse peroxidase-conjugated antibodies were from Cell Signaling Technology (Beverly, MA, USA). Pharmalyte, pH 3-10, and ECL® Advance reagent were obtained from GE Healthcare (Buckinghamshire, UK). Bio-Rad protein assay dye reagent was purchased from Bio-Rad (Hercules, CA, USA). 3-(4,5-dimethylthiazol-2-yl)-2,5-diphenyltetrazolium bromide (MTT), 2’,7’-dichlorodihydrofluorescein diacetate (DCFH-DA), 2,2-azinobis-(3-ethylbenzothiazoline-6-sulfonic acid) (ABTS), bovine serum albumin (BSA), reduced glutathione (GSH), dimethyl sulfoxide (DMSO), DMEM F12, fetal calf serum (FCS), horse serum (HS), Gentamicin, Amphotericin B, anti-β-actin, and all other chemicals of the highest analytical grade were purchased from Sigma-Aldrich (Milan, Italy). 3,8-phenanthridinediamine, 5-(60-triphenylphosphoniumhexyl)-5,6 dihydro-6-phenyl (MitoSOX) was purchased from Invitrogen (Paisley, UK). Dihydroethidium (DHE) was purchased from Molecular Probes (Eugene, OR, USA).

### Ethics Statement

The investigation conforms with the Guide for the Care and Use of Laboratory Animals published by the US National Institutes of Health (NIH Publication No. 85-23, revised 1996) and approved by the Ethics Committee of our Institution (Comitato Etico Scientifico per la Sperimentazione Animale, Alma Mater Studiorum - Università di Bologna). 

### Cell culture and treatments

Neonatal Wistar rat cardiomyocytes were isolated as previously reported [20]. Briefly, cells were obtained by isolation of cardiomyocytes from the ventricles of 2–4 days old Wistar rats, seeded at a density of 1x10^5^ cells/cm^2^ and grown until confluence in DMEM F12 supplemented with 10% v/v FCS and 10% v/v HS. Cells were treated with 5 μM SF for different time points, and control cells were treated with equivalent concentrations of DMSO alone. SF concentration utilized in this study is readily achievable in rat and human plasma [21,22] and is close to that achievable in human plasma after a single portion of broccoli sprout isothiocyanate intake [23]. 

### Protein extraction

SF-treated and control cells were washed twice with cold PBS and twice with 10 mM Tris-HCl/250 mM sucrose pH 7.4 to remove salts. Cells were lysed in rehydration buffer, incubated for 1 h at room temperature and stored at -80°C. After thawing, samples were centrifuged at 18,000 *g* at 4°C for 15 min and the supernatant was immediately used or aliquoted and frozen at -20°C. Protein concentration was determined according to the Bradford method [24]. 

### Two-dimensional gel electrophoresis and image analysis

At confluence, cardiomyocytes were treated with 5 µM SF or the DMSO vehicle control for 1, 6, 24 and 48 h. Isoelectric focusing (IEF) was carried out on Immobiline DryStrips (GE Healthcare, Buckinghamshire, UK), 13 cm long, with a pH 3-10 linear gradient, using an IPGphor apparatus (GE Healthcare). For analytical runs: IPG strips were rehydrated with 200 μg of protein in 250 μL of IPG rehydratation solution (8 M urea, 2% CHAPS, 10 mM DTT, 1% v/v Pharmalyte and trace bromophenol blue) for 12 h at 50 V. The strips were then focused according to the following voltage steps: 1 h at 500 V, 1 h at 1000 V, 30-min ramp up to 8000 V, and 2 h at 8000 V until a total of 19 kVh was reached.

For mass spectrometry (MS)-preparative runs: IPG strips were overnight rehydrated in 8 M urea, 4% CHAPS, 1% DTE, 1% v/v Pharmalyte, and a trace of bromophenol blue. IEF was performed by applying 1000 μg of protein using the sample cup loading system (GE Healthcare) at the cathodic end of strips. Running conditions were as follows: 200 V for 24 h, 500 V for 30 min, 1250 V for 1 h, from 1250 to 3500 V in 1 h, 3500 V for 3 h, from 3500 to 5000 V in 1 h, 5000 V for 6 h, from 5000 to 8000 V in 2 h, and 8000 V until reaching a total of 100 kVh.

Prior to the second dimension run, analytical and preparative gel strips were equilibrated sequentially in two equilibration buffered solutions (50 mM Tris-HCl pH 6.8, 6 M urea, 30% glycerol, 2% SDS), one containing 2% DTE, and the other 2.5% iodoacetamide. SDS-PAGE was carried out on 15% acrylamide gels at 30 mA/gel using a Protean II xi Cell (Bio-Rad) at 10°C, until the dye front reached the bottom of the gel. 2-DE analytical gels were stained by ammoniacal silver nitrate, as previously described [25,26], while the preparative gels were stained applying a MS-compatible silver staining protocol [27]. Analytical and MS-preparative gels were then digitalized using a GS-800 imaging densitometer (Bio-Rad).

At least three biological replicates with two technical replicates were run for each tested condition and the resulting protein maps were analyzed by PDQuest v 8.0.1 software (Bio-Rad). Normalized spot volume values (Vol = integration of OD over the spot area; Normalized Vol = Vol of a single spot divided by the total spot Vol) were used for statistical evaluation of protein abundance. The coefficient of variation (CV, calculated by the standard deviation of the normalized spot volumes divided by the mean, expressed as a percent) and the correlation coefficient, as r^2^, of the normalized spot volumes were calculated as a measure of within- and between-sample variance

### In-gel tryptic digestion and MS protein identification

Spots of interest were manually excised from MS-preparative 2-DE gels, destained and acetonitrile (ACN) dehydrated. Successively, they were rehydrated with a trypsin solution (Sigma-Aldrich) and in-gel digestion was carried out overnight at 37°C. Protein digests (0.75 µl for each spot) were spotted onto a MALDI-TOF target (GE Healthcare) and allowed to dry. Then, 0.45 µl of matrix solution (saturated solution of α-cyano-4-hydroxycinnamic acid in 50% v/v ACN and 0.5% v/v trifluoroacetic acid (TFA)) was applied to the dried sample, dried again and tryptic peptide masses were acquired on an Ettan MALDI-ToF Pro mass spectrometer (GE Healthcare). Mass spectra were acquired in reflectron mode with an accelerating voltage of 20 kV using the Ettan MALDI Evaluation software (GE Healthcare). Peptide masses were internally calibrated using the 842.509 and 2211.105 *m/z* trypsin autolysis peptides. All the obtained mass lists were automatically cleaned up from eventually present contaminant masses, such as those from matrix, auto-digestion of trypsin, and keratins. Protein identification was carried out by peptide mass fingerprinting searching in UniProtKB (SwissProt 2012_08: 537505 sequences (*Rattus*: 7800 sequences); 190795142 residues) and NCBInr (NCBInr 20120922: 20543454 sequences (*Rattus*: 68040 sequences); 7050788919 residues) databases using Mascot (Matrix Science Ltd., London, UK, http://www.matrixscience.com) on-line-available software. Searches were performed allowing a mass tolerance of 100 ppm and a single missed cleavage site, with carbamidomethyl cysteine as fixed modification and oxidized methionine as variable modification. The taxonomy was limited to *Rattus norvegicus*, whereas no restrictions on protein molecular weight and pI values were applied. The criteria used to accept identifications included the extent of sequence coverage, number of matching peptides, and probabilistic score sorted by the software, as detailed in Table S1.

In the case of ambiguous identifications, tryptic digests were subjected to ESI-Ion trap MS/MS peptide sequencing on a nanospray/LCQ DECA Ion Trap mass spectrometer (Thermo Scientific, San Jose, CA, USA). Peptide samples were acidified with 1% TFA, concentrated and desalted using mC18 Zip-Tips (Millipore, Billerica, MA, USA). Tryptic peptide elution was achieved with a 70% methanol and 0.5% formic acid solution and concentrated samples were loaded in a distal coated Fused-Silica Picotip emitter to be nanosprayed in the mass spectrometer. The collision energy was set based on the mass of the doubly charged precursor ions and spectra were recorded using Xcalibur software (Thermo Scientific). Database search of MS/MS spectra was performed by TurboSEQUEST (Thermo Scientific) and Mascot MS/MS ion search software (http://www.matrixscience.com) in UniProtKB and NCBInr non-redundant databases. The following criteria were applied: MS mass accuracy ± 1.2 Da, MS/MS mass accuracy ± 0.6 Da, peptide precursor charge 2+, monoisotopic experimental mass values, trypsin digestion with one accepted missed cleavage, fixed carbamidomethylation of cysteine and variable oxidation of methionine.

### Bioinformatic analysis of the proteomic data

Only “reliable” protein spots were considered (i.e. given *n* experimental replications per condition, a protein spot was kept if at least (*n*-1) volume values were available for all samples) and missing values were imputed using the k-nearest neighbor (KNN) method (Matlab v 7.12.0, MathWorks, Torino, Italy). Pairwise comparisons of protein spot abundances were performed using Student’s *t*-test (SigmaStat v 3.5 software, Systat Software, Point Richmond, CA, USA). A p value <0.05 was considered as statistically significant. Multivariate data analysis methods were used for selecting significant spots and classifying the different group samples. Principal Component Analysis (PCA) was carried out using CANOCO for Windows v 4.5. All variables were centered and weighted by (standard deviation)^-1^.

Significant differences were analyzed through the two-way hierarchical clustering methodology using PermutMatrix v 1.9.3 software (http://www.lirmm.fr/~caraux/PermutMatrix/). Samples were clustered employing the Pearson and Ward’s minimum variance methods for distance and aggregation, respectively.

Identified proteins were annotated using the publicly available on-line Gene Ontology (GO) database, AmiGO version 1.8 (database release 31-03-2012; http://amigo.geneontology.org), which standardizes the representation of genes and their products by using a controlled vocabulary in terms of location, biological processes and molecular function. Protein accession numbers and their corresponding fold changes were imported into the web-based integrative software MetaCore^TM^ (v 6.8 build 30387; Thomson Reuters, St. Joseph, MI, USA) for enrichment and network analysis. The enrichment analysis of GeneGo pathway maps, metabolic networks and process networks was based on hypergeometric distribution statistics. The network building of the dysregulated proteins was carried out using the shortest path algorithm and its variants. Networks were ranked according to their statistical significance (p<0.001) and interpreted in terms of GO. Major hubs were delineated based on the connections and edges within the networks.

### Functional analysis of proteomic data

Protein accession numbers of the MS-identified differentially expressed proteins and their corresponding fold changes were imported into the web-based integrative software MetaCore^TM^ (v 6.8 build 30387; Thomson Reuters, St. Joseph, MI, USA) for enrichment and network analysis. For detailed information see Supplementary data online.

### Western blot analysis

Differential expression of CLP36, GLO1 and MIF was validated by western blotting. Protein samples were resolved in 10% acrylamide gels and then transferred onto nitrocellulose membranes in a trans-blot electrophoresis transfer cell (Bio-Rad). The following primary antibodies were used: anti-CLP36 (1:500), anti-GLO1 (1:500), anti-MIF (1:1000), and anti-β-actin (1:5000) (Santa Cruz Biotechnology, Santa Cruz, CA, USA). 

### Assessment of cell viability and apoptosis

Cell viability was evaluated by measuring 3-(4,5-dimethylthiazol-2-yl)-2,5-diphenyltetrazolium bromide (MTT) reduction as previously reported [28]. Briefly, at the end of each experiment, 0.5 mg/ml MTT was added and incubated for 1 h at 37°C. After incubation, MTT solution was removed, 200 µl DMSO was added, and the absorbance was measured at λ=595 nm using a microplate spectrophotometer (VICTOR3 V Multilabel Counter; Perkin-Elmer, Wellesley, MA, USA).

### Detection of Intracellular Reactive Oxygen Species levels (ROS)

#### Confocal microscopy

ROS formation in cardiomyocytes was visualized as follows. Cells were incubated with 5 μM SF for 24 h, exposed to1 mM MG for 24 h, and then washed twice with warm PBS, and DHE and MitoSOX were added to cells at a final concentration of 5 µM. After incubation with DHE (20 min) and MitoSOX (10 min), cells were fixed with 4% paraformaldehyde for 10 min, mounted onto glass slides with Mowiol, and observed under a confocal microscope with excitation and emission wavelengths set to 490 and 590 nm, respectively [29,30]. All images were taken under identical exposure conditions in order to evaluate the intensity of the probe fluorescence accurately. Confocal imaging was performed on a Nikon A1 confocal laser scanning microscope as previously described [31]. The confocal serial sections were processed with ImageJ software to obtain three-dimensional projections, as previously described [32]. The image rendering was performed by Adobe Photoshop software.

#### Spectrofluorimetric assays

ROS producion was evaluated using different fluorescent probes: DCFH-DA, DHE and MitoSOX. Briefly, controls and SF-treated cells were washed with PBS and then incubated with 5 μM DCFH-DA for 30 min. Next, cells were incubated with 1 mM MG. Cell fluorescence was measured using a microplate spectrofluorometer (VICTOR3 V Multilabel Counter) (λex/em=485/535 nm) as previously reported [24]. Cells were treated with 5 μM SF for 24 h and then exposed to 1 mM MG. Cells were washed with PBS and incubated with 5 μM DHE for 20 min or 5 μM MitoSOX for 10 min at 37 °C. Cell fluorescence was measured using a microplate spectrofluorometer (VICTOR3 V Multilabel Counter) (λex/em=485/590 nm).

#### Total antioxidant activity (TAA)

TAA assay, performed as previously reported [33], was used as an indirect index of intracellular ROS production. Briefly, at the end of each experiment, cardiomyocytes were washed 3 times with cold PBS. Cells were lysated in PBS using a potter homogeniser and centrifuged at 1000 g to remove cell debri. TAA was determined by the decoloration of the radical cation ABTS, in terms of quenching of absorbance at 740 nm. Values obtained for each sample were compared with the concentration–response curve of a standard Trolox solution, and expressed as µmol of Trolox Equivalent Antioxidant Activity per mg of protein (TEAA µmol/mg protein).

### Caspase-3 activity assay

Caspase-3 activity was measured by means of a fluorimetric assay based on the hydrolysis of the peptide substrate acetyl-Asp-Glu-Val-Asp-7-amido-4-methylcoumarin (Ac-DEVD-AMC) by caspase-3, resulting in the release of the fluorescent 7-amino-4-methylcoumarin (AMC) moiety as previously reported [28]. Briefly, cells received 5 µM SF for 24 h and were subsequently treated with 1 mM MG for further 24 h. At the end of the treatment cells were washed twice with ice-cold PBS, scraped off and incubated on ice in lysis buffer (50 mM HEPES pH 7.4, 5 mM CHAPS and 5 mM DTT) for 20 min. Samples were centrifuged at 15 000 *g* for 15 min at 4°C and protein concentration in supernatants was determined according to the Bradford method [24]. 

From each sample, 5 µl was transferred to a 96-well plate and 200 µl of reaction mix (20 mM HEPES pH 7.4, 0.1% CHAPS, 5 mM DTT and 2 mM EDTA) containing Ac-DEVD-AMC (final concentration 16 µM), was added to each well. Fluorescence intensity was recorded every 5 min for 1 h by a microplate spectrofluorometer (λ_ex/em_= 360/460 nm). Caspase activity was calculated using a AMC standard curve and results were expressed as nmol AMC/min/mg protein.

### GLO1 activity assay

Cardiomyocyte GLO1 activity was assessed by spectrophotometry according to the method of McLellan and Thornalley [34] with minor modifications. Briefly, cells were lysed in 10 mM PBS and protein concentrations were estimated using the Bradford’s method [24], GLO1 activity assay was performed by addition of 20 μl of cell lysate to a reaction mixture containing 2 mM MG and 2 mM GSH, which had been equilibrated for 10 min before sample addition.

### AGE assay

AGE protein adducts were quantified by the OxiSelect™ AGE ELISA Kit (Cell Biolabs, San Diego, CA, USA) according to manufacturers’ instructions.

### Statistics

Each experiment was performed at least three times, and all values are represented as mean ± SD. Statistical analysis was performed with Student’s *t*-test and one-way ANOVA followed by Dunnett’s or Bonferroni’s test (Prism 5; GraphPad Software, San Diego, CA, USA). Statistical significance was considered to be achieved when p<0.05. For statistical analysis of proteomic data see Supplementary data online.

## Results

### Time-course proteomic profiling of SF-treated cardiomyocytes

**Figure 1:**
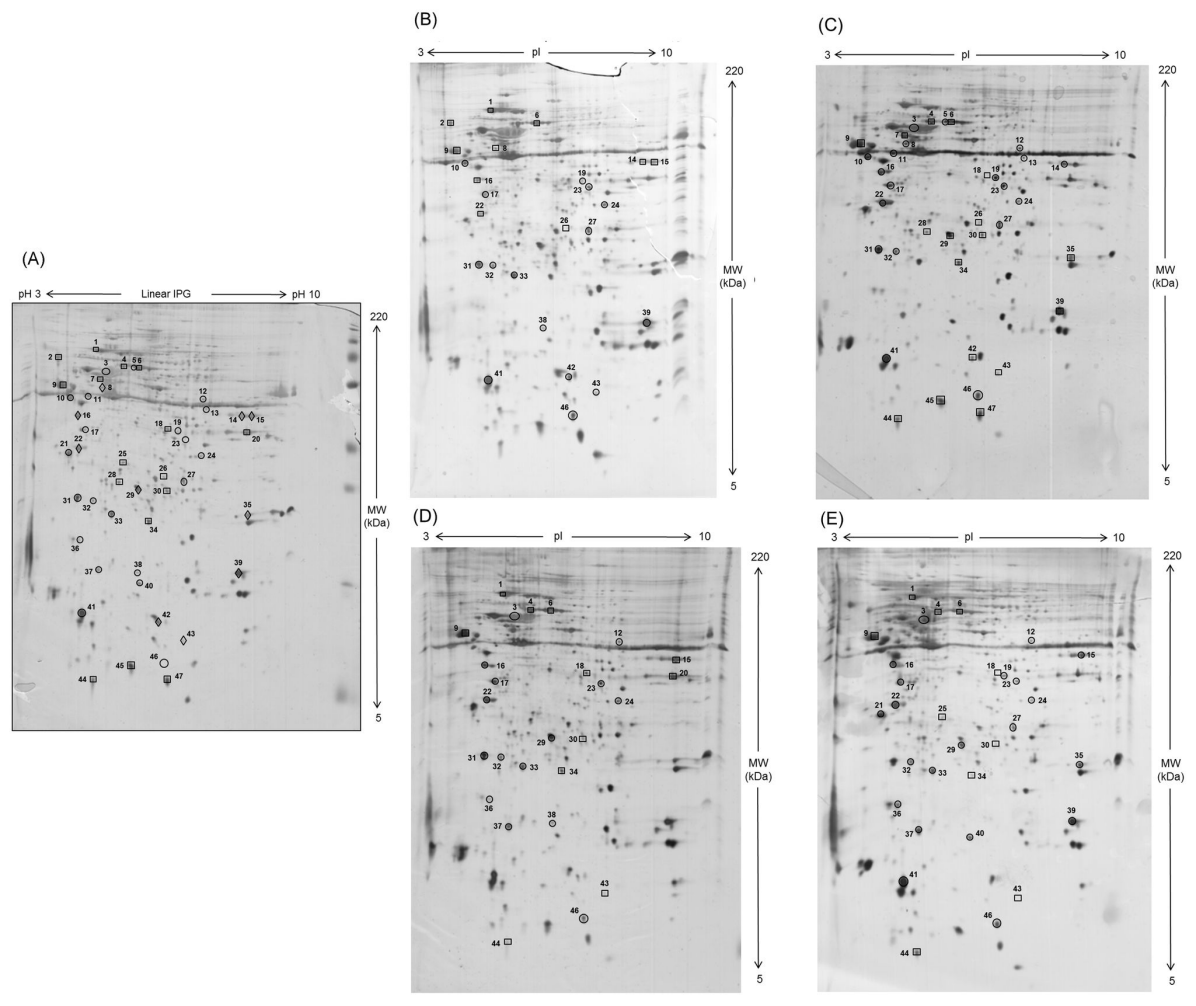
2-DE protein profile of cardiomyocytes treated with SF. IEF performed on IPG strips (13 cm, 3-10 linear pH gradient) was followed by second dimension separation on a 15% polyacrylamide gel. 2-DE gel was silver-stained. Identified spots, showing a statistically significant difference of at least 1.5-fold between SF-treated cardiomyocytes at different time points [1 (B), 6 (C), 24 (D) and 48 (E) h] and vehicle control (A), are numbered (Table S1). Circles and squares indicate up- and down-regulated protein spots, respectively. (A) Diamonds indicate protein spots with opposite trends of expression among the time points of exposure to SF.

**Figure 2:**
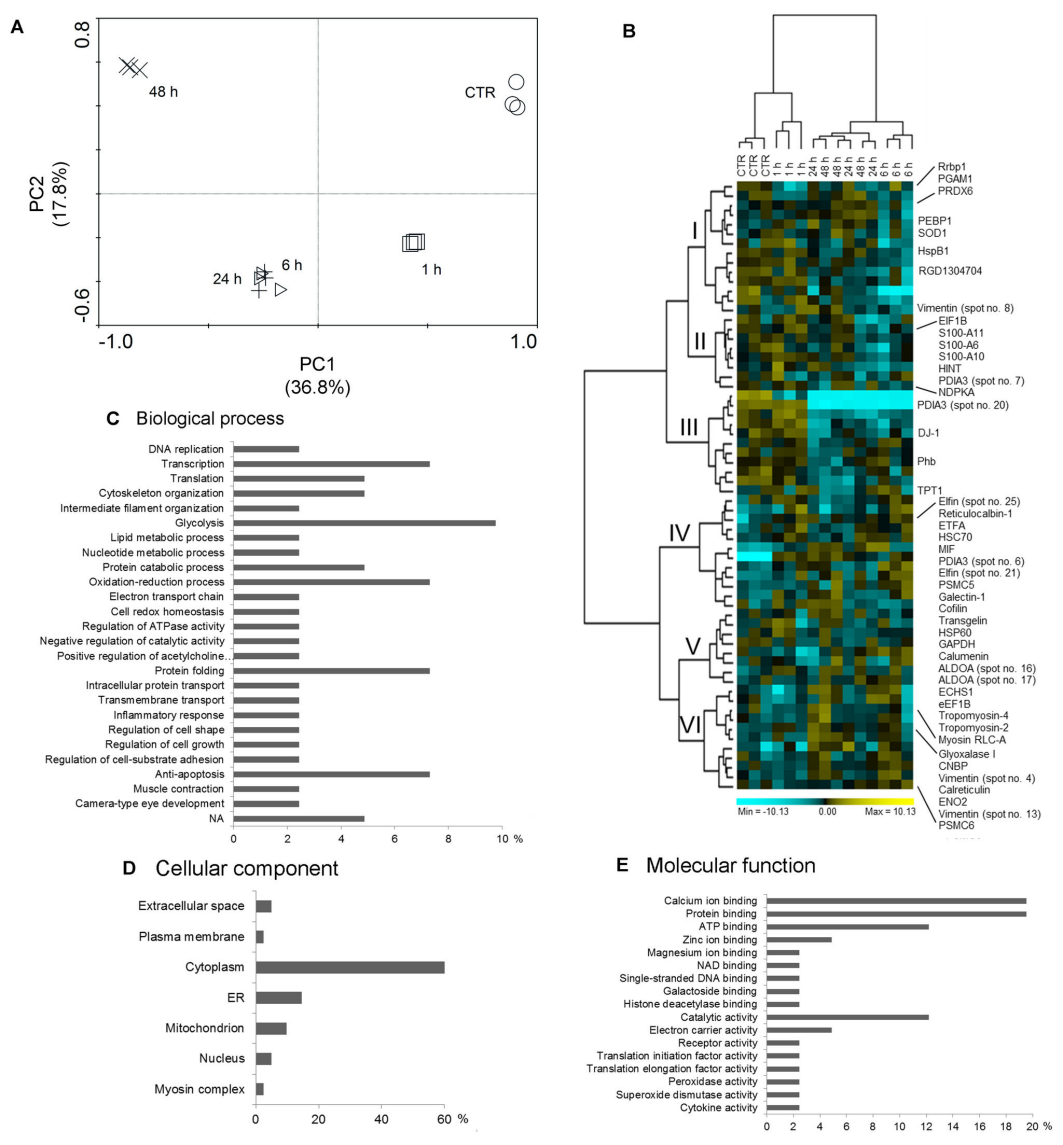
Multivariate analysis of proteomic data. (**A**) PCA plot of the protein spot maps of cardiomyocytes untreated (DMSO vehicle control; circles) and after exposure to SF for 1 (triangles), 6 (squares), 24 (diamonds) and 48 h (crosses). Log-transformed data were used. Each symbol represents a 2-DE gel from each treatment series. First and second ordination axes are plotted explaining 36.8 and 17.8% of the overall variance in the dataset, respectively. (**B**) Two-way hierarchical clustering of the 64 differentially expressed protein spots between SF-treated cardiomyocytes and DMSO vehicle control. Pearson's dissimilarity as distance measure and Ward's method for linkage analysis were used. Log_2_ ratios are colour coded as indicated. Names of the identified protein spots are shown on the right (see Table S1). A larger version of this image with gene names and spot IDs in brackets is available as Figure S1. (**C**-**E**) Functional categories by Gene Ontology (GO) analysis of the identified proteins. Classification was performed according to keyword categories [(**C**) biological process, (**D**) cellular component, (**E**) molecular function]. When proteins were associated with more than one functional category, one GO term was chosen arbitrarily. NA, not available. ER, endoplasmic reticulum.

To gain insights into the molecular pathways modulated by SF, time-dependent differential protein expression profiles of cardiomyocytes treated with SF were investigated. Cardiomyocytes were exposed to 5 µM SF for 1-48 h, and for each sampled time point, protein extracts were resolved applying 2-DE. Cardiomyocytes treated with 0.05% DMSO (vehicle control) were used as a control. Representative silver-stained 2-DE gels of DMSO- and SF-treated cardiomyocytes are shown in Figure 1. In each gel more than 800 spots were detected, quantified, normalized and inter-gel matched. The mean CV for normalized spot volumes was determined to be 18% for the analytical variance and 41% for the biological variance. The mean r^2^ values for within-sample comparisons ranged from 0.78 to 0.88. The r^2^ values for between-sample comparisons were 0.72 to 0.80. The entire 2-DE dataset was processed using the unsupervised multivariate method PCA. PCA revealed a generally consistent reproducibility between the biological replicates and a non-linear behavior during the time course (Figure 2A). The first two PCs accounted for about 55% of the total variance in the data (36.8 and 17.8% for PC1 and PC2, respectively). Interestingly, the protein patterns of 1 h SF-treated cardiomyocytes were clearly segregated from DMSO control, suggesting that significant changes in protein expression occurred early after SF exposure. Conversely, spot maps at 6 and 24 h were almost completely overlapped, indicating that no additional differences in the SF-treated cardiomyocyte proteome occurred during this time interval. After 48 h, a clearly different protein expression profile was evidenced.

In addition to multivariate analysis, pair-wise comparisons of individual protein spots were performed using Student’s *t*-test and the non-parametric Mann-Whitney test. Protein expression changes were considered significant when relative spot abundance increased or decreased by at least 1.5-fold between DMSO control and the different time points with p<0.05. Sixty four significant variations were found. Differential spots were clustered using Ward’s minimum variance method over a Pearson distance-based dissimilarity matrix. As shown in Figure 2B and S1, spot maps were generally partitioned according to the experimental sample set, except for the overlaps between the expression profiles at 24 and 48 h. Two main clusters were recognized, each subdivided in at least 3 sub-clusters. In particular, clusters I and II included 14 and 8 proteins, respectively, whose abundance dramatically decreased after 6 h SF treatment before returning to baseline levels at later time points. Analogously, the 11 cluster III proteins were up-regulated in DMSO- and 1 h SF-treated cardiomyocytes, but down-regulated at 6 h. Conversely, the other three clusters (IV-VI) grouped 12, 8 and 11 protein spots, respectively, whose expression levels increased over time after SF treatment.

Following Mascot database search using acquired MS data, 47/64 (73%) protein spots were successfully identified, corresponding to 41 unique proteins. Details of the protein identification, protein score, sequence coverage, theoretical/experimental pI value and molecular weight as well as average relative change at each treatment time point are summarized in Table S1. Representative spectra and mass lists for three identified protein spots are shown in Figure S2. Identified proteins were categorized according to ontological categories (http://amigo.geneontology.org; Figure 2C,D,E and Table S2). The most represented biological processes were transcription/translation (12%), oxidation-reduction process/cell redox homeostasis (10%), glycolysis (10%), protein folding (7%), and anti-apoptosis (7%). More than half of the SF responsive proteins (61%) were cytoplasmic. Endoplasmic reticulum accounted for 15% of the identifications, whereas mitochondrial proteins were 10%. According to molecular function, as much as 68% of the significantly modulated proteins were classified as binding proteins. In particular, nearly one-third of affected proteins were involved in calcium ion and protein binding. Twelve percent were related to catalytic activity. Expression profiles of proteins comprised in Figure 2E are shown in supplementary material (Figure S3).

Pathways and networks involving the differentially expressed proteins were analyzed using MetaCore. Enrichment analysis revealed that the most enriched pathways (Figure S4A) and metabolic networks (Figure S4B) were related to glycolysis and gluconeogenesis. Additionally, enrichment in the ontology of functional processes, as defined by “GeneGo process networks”, was calculated. Regulation of cytoskeleton rearrangement and protein folding resulted as top-scoring networks (Figure S4C). 

**Figure 3:**
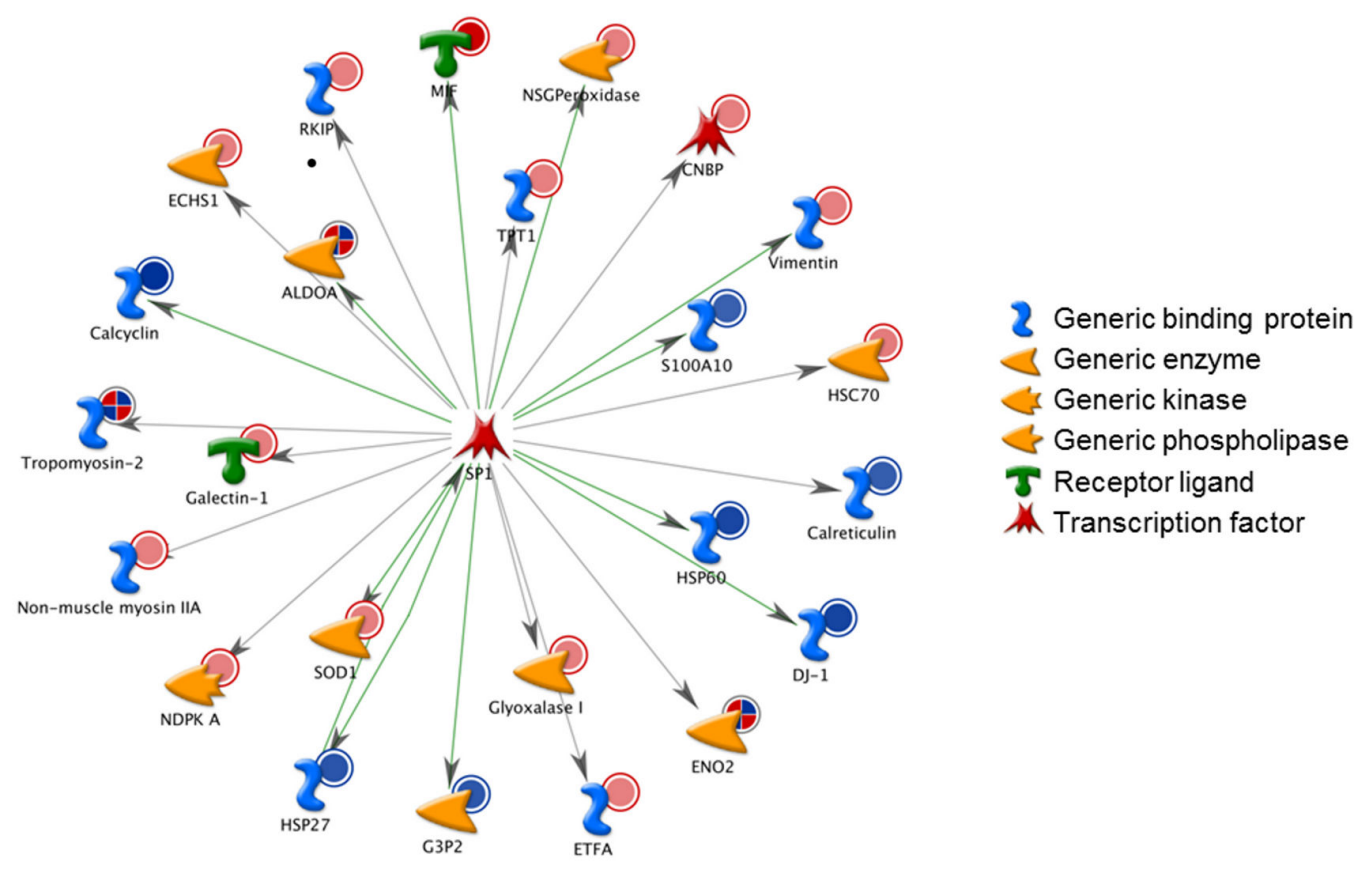
Network analysis of differentially expressed proteins. The top score transcription regulation network initiated through activation of SP1 is shown. Network proteins are visualized by proper symbols, which specify the functional nature of the protein (network caption). Green and gray arrows indicate positive and unspecified effects, respectively. Red and blue circles indicate up- and down-regulated proteins following SF treatment, respectively. ‘Checkerboard’ color indicates mixed expression between files.

To map interaction between proteins, the shortest direct paths were performed using the “analyze network” algorithm. Based on the functional sub-networks built, the SF responsive proteins were primarily involved in signaling pathways (p = 5.13×10^-26^), as well as regulation of immune response (p = 2.89×10^-8^) (Table S3). Then, to identify specific transcription factors which could drive the proteome changes in SF-treated cardiomyocytes, a variant of the “shortest paths” algorithm was used to generate transcriptional regulation networks. SP1 was ranked #1, with 24 targets among the 41 identified proteins from 2-DE gels (Figure 3). The regulation mechanisms of 13/24 SP1 targets were unknown. Other top-scoring transcriptional regulators were c-Myc and p53 (Table S4).

### Validation of time-dependent differential expression of MIF, CLP36 and GLO1

Validation of proteomics findings by western blot analysis was performed to confirm the differential expression of MIF, CLP36 and GLO1. These proteins were selected for both their increased expression after SF treatment and their possible involvement in SF-induced cardioprotection [12,17]. In particular, it has recently been shown that MIF provides cardioprotection during I/R by reducing oxidative stress [35], that CLP36 plays an important role in heart development [36], and that GLO1 is an enzymatic defence against α-oxoaldehyde-mediated glycation [13].

**Figure 4:**
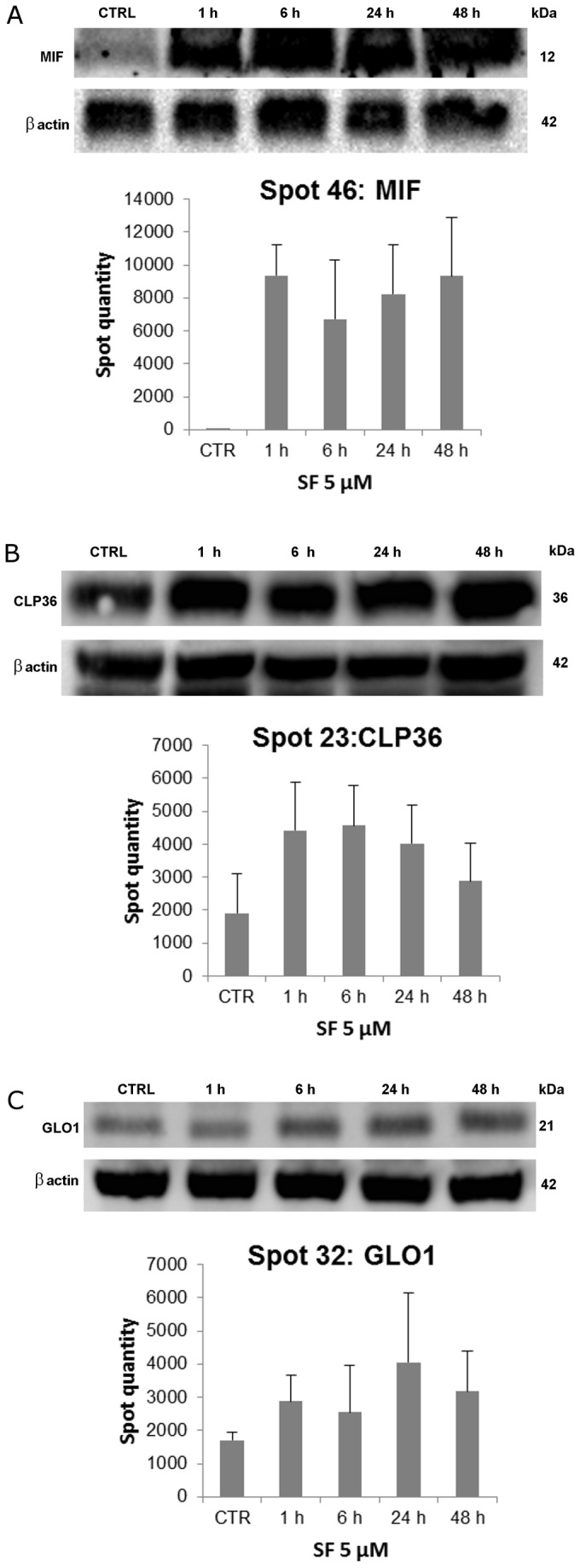
Validation of MS results by immunoblotting. Cardiomyocytes were treated with 5 μM SF for 1-48 h followed by western blot analysis for MIF (**A**), CLP36 (**B**), and GLO1 (**C**). Representative bands and raw densitometric values of the corresponding protein spots from proteomic analysis are shown. Each bar represents the spot quantity as determined by PDQuest (mean ± SD). At least three biological replicates with two technical replicates were run for each tested condition.

Representative immunoblots of MIF, CLP36 and GLO1 together with densitometric quantifications of the spots obtained by proteomic analysis are shown in Figure 4. Consistent with 2-DE maps, the expression of MIF was markedly elevated already after 1 h SF treatment. Similar results were obtained for CLP36, while GLO1 expression was higher than DMSO control after 6 h treatment. All these increases in protein level lasted until 48 h after SF addition.

### SF protection against MG-induced damage

**Figure 5:**
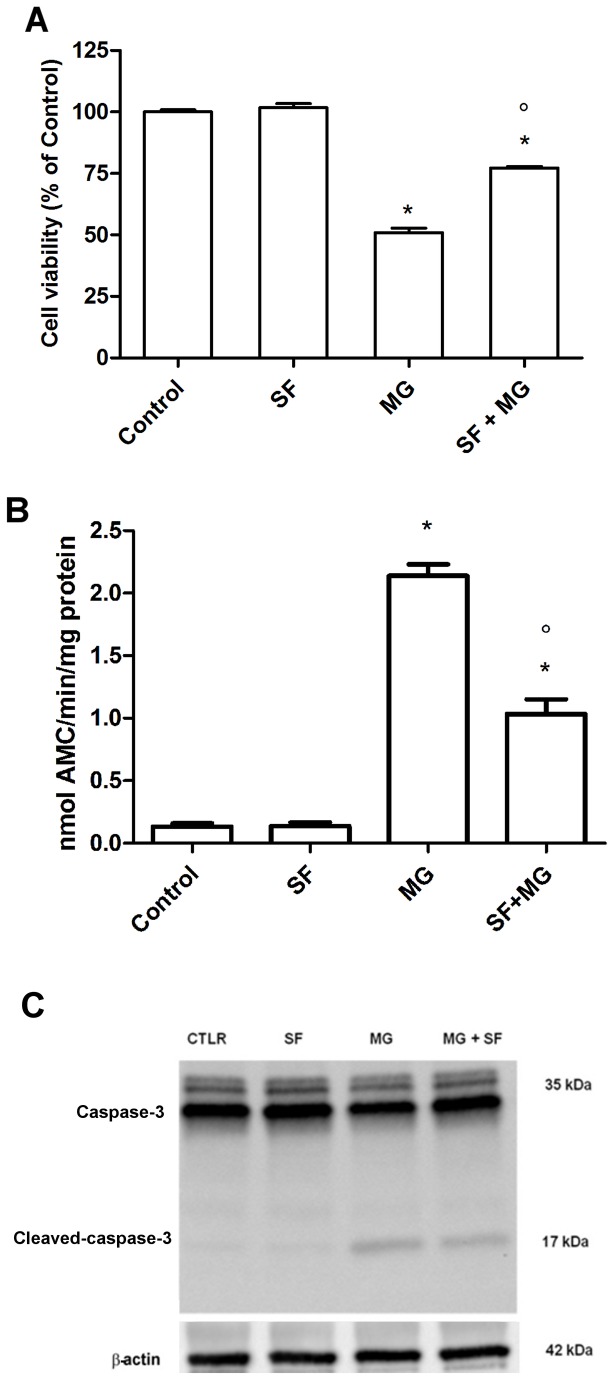
SF protection against MG-induced damage. Cardiomyocytes were treated with 5 µM SF for 24 h before addition of 1 mM MG. (**A**) Cell viability was assessed by MTT assay and reported as percent cell viability in comparison to control. (**B**) Caspase-3 activity was measured spectrofluorimetrically by hydrolysis of the peptide substrate acetyl-Asp-Glu-Val-Asp-7-amido-4-methylcoumarin (Ac-DEVD-AMC). (**C**) Cell lysates were immunoblotted with caspase-3 antibody that detects both full length caspase-3 (35 kDa) and the large fragment of caspase-3 resulting from cleavage (17 kDa). Data are presented as mean ± SD, n = 4 in each group,* p<0.05 vs Control; ° p<0.05 vs MG.

To better understand the role of SF-induced GLO1 up-regulation, we studied the protective effects of SF against MG, the main GLO1 substrate, in cardiomyocytes. First of all, IC_50_ for MG cytotoxicity was determined. Cardiomyocytes were treated with increasing concentration of MG (0.1-5 mM) and the MTT viability assay was performed after 24 h (Figure S5). One mM MG was considered as IC_50_ value and used in the subsequent experiments. To evaluate the ability of SF to protect cardiomyocytes from MG-induced damage, cells were pre-treated with 5 µM SF for 24 h and subsequently exposed to 1 mM MG for further 24 h before MTT viability assay. As shown in Figure 5A, SF was able to counteract MG-induced damage, significantly increasing cell viability with respect to MG. Moreover, caspase-3 activity assay and immunoblotting analysis of caspase-3 expression revealed that SF markedly protected cardiomyocytes against apoptosis induced by MG. In figure 5B caspase-3 activity in cardiomyocytes pre-treated with 5 µM SF for 24 h and then exposed to 1 mM MG for further 24 h is reported. MG strongly increased caspase-3 activity compared to control cells, evidencing a strong MG-induced apoptotic cell death, whereas SF pre-treatment significantly reduced this increase. These data were confirmed by immunoblotting of caspase-3 (Figure 5C), which showed the appearance of the activated caspase-3 fragment and the corresponding decrease of pro-caspase-3 after MG treatment. SF pre-treatment reduced the protein level of activated caspase-3 with respect to MG-treated cells.

**Figure 6:**
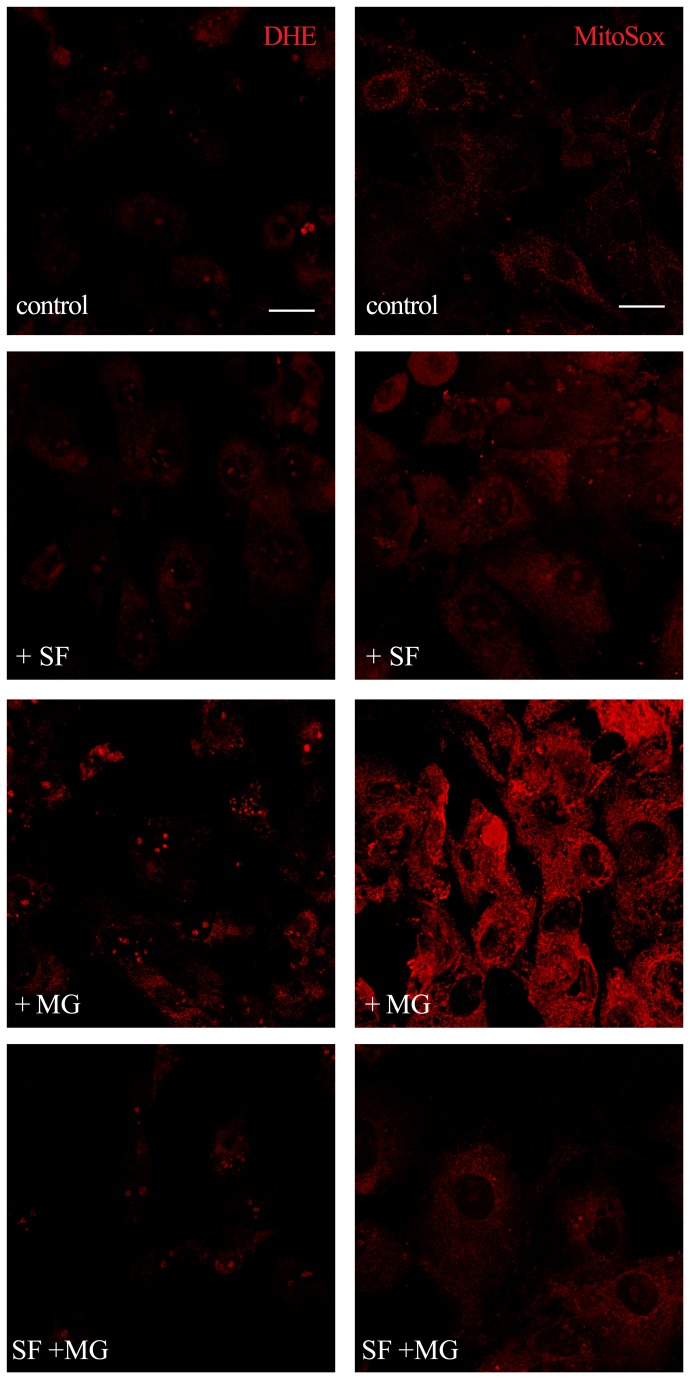
Confocal microscopy analysis of SF effect on MG-induced ROS production. Cardiomyocytes were treated with 5 µM SF for 24 h before the addition of 1 mM MG. Cytosolic ROS production was measured using DHE and mitochondrial ROS production by MitoSOX staining. Scale bar: 10 µm.

**Figure 7:**
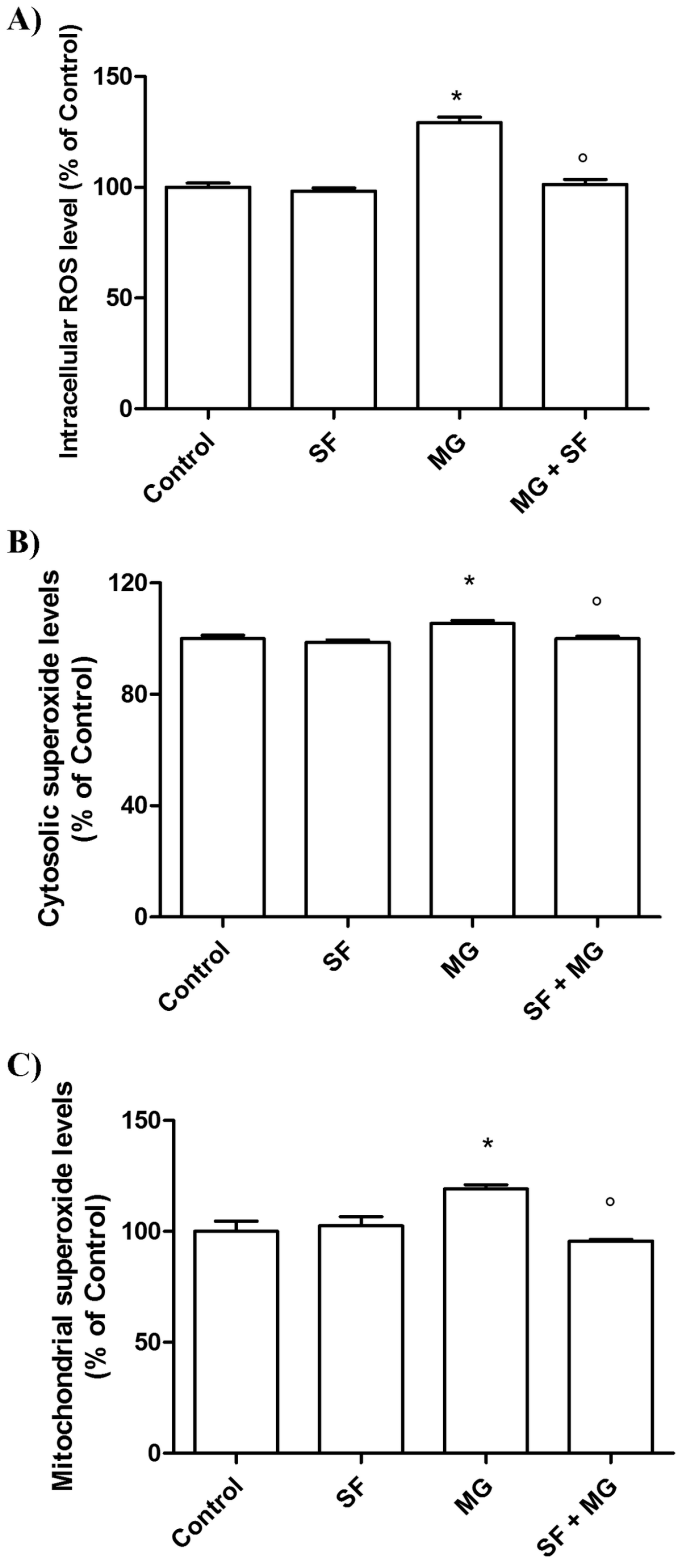
Fluorimetric assays of SF effect on MG-induced ROS generation. Cardiomyocytes were treated with 5 µM SF for 24 h before addition of 1 mM MG. (**A**) Intracellular ROS levels were determined with the peroxide-sensitive probe DCFH-DA. (**B**) Cytosolic ROS levels were determined with DHE. (**C**) Mitochondrial ROS levels were determined with MitoSox. Data are expressed as a percentage compared to control and are presented as mean ± SD, n = 4 in each group,* p<0.05 vs Control; ° p<0.05 vs MG.

GLO1 is a GSH-dependent enzyme and excess MG presents serious toxic effects since it depletes GSH via a covalent bond. As GSH is one of the most important endogenous antioxidant molecules, its depletion could be related to an increase in ROS. To verify this hypothesis we used 3 different probes, DCHF-DA, DHE, and MitoSOX as it is widely accepted that a single readout for ROS generation is not appropriate [37]. Representative confocal fluorescent micrographs of cardiomyocytes pre-treated with 5 µM SF for 24 h, exposed to 1 mM MG and stained with DHE or MitoSox are shown in Figure 6. MG induced a marked increase in DHE and MitoSox fluorescence intensity, while SF was able to maintain fluorescence intensity to value comparable to control cells. SF alone had no effect on fluorescence intensity of cells stained with DHE and MitoSox. Semi-quantitative measurements by fluorescent plate reader of DCHF-DA, DHE and MitoSox stained cardiomyocytes are reported in Figure 7. MG exposure induced a significant increase in intracellular ROS levels. In particular, the highest increase was obtained with DCHF-DA, the less specific probe able to detect not only H_2_O_2_ but also several one-electron-oxidizing species [37] (Figure 7A). In agreement with the results obtained by confocal microscopy, MG treatment significantly increased the fluorescence intensity of DHE and MitoSox that more specifically target superoxide (Figure 7B and C). SF was able to counteract the intracellular ROS level increase induced by MG, maintaining fluorescence intensity of the 3 probes to levels comparable to control cells.

**Figure 8:**
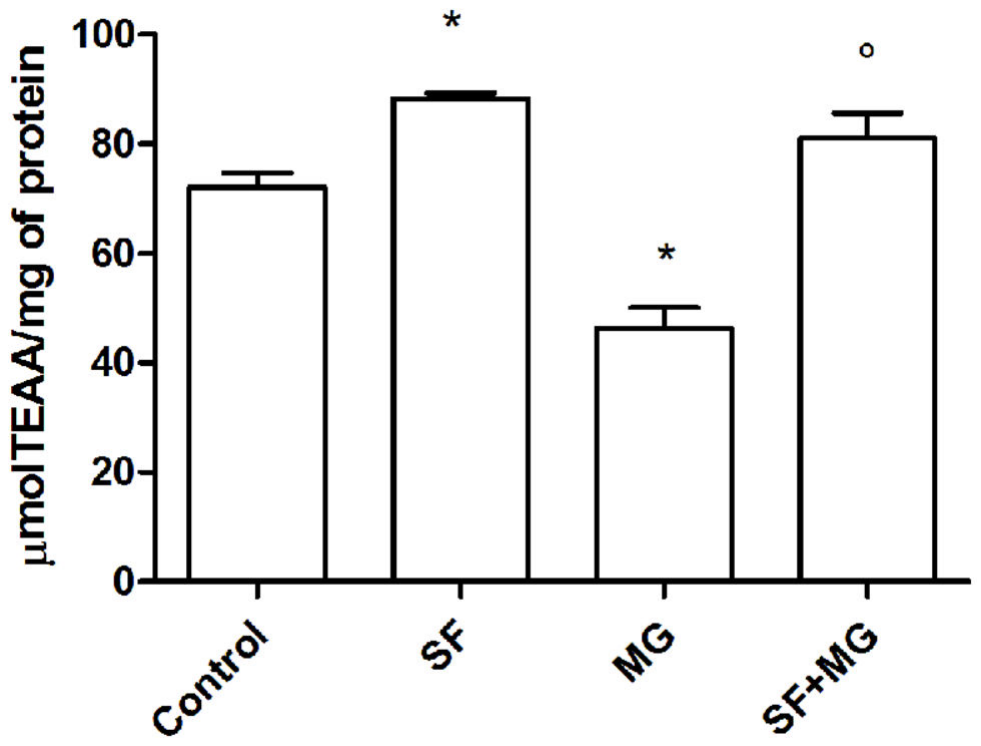
SF effects on cardiomyocytes total antioxidant activity. Cardiomyocytes were treated with 5 µM SF for 24 h before addition of 1 mM MG. Cell lysates were submitted to the ABTS radical cation decolorization assay and the antioxidant activity of the cells were expressed as mean ± SD of µmol of trolox antioxidant activity per mg of protein. p<0.05 vs Control; ° p<0.05 vs MG.

To better elucidate SF role in counteracting oxidative stress, total antioxidant activity (TAA) by ABTS assay was evaluated (Figure 8). SF alone was able to significantly increase TAA in respect to control cells, while MG significantly reduced this value. Interestingly, SF was able to restore TAA levels of MG treated cardiomyocytes to values comparable to control cells.

### Effect of SF against MG-induced glycation

**Figure 9:**
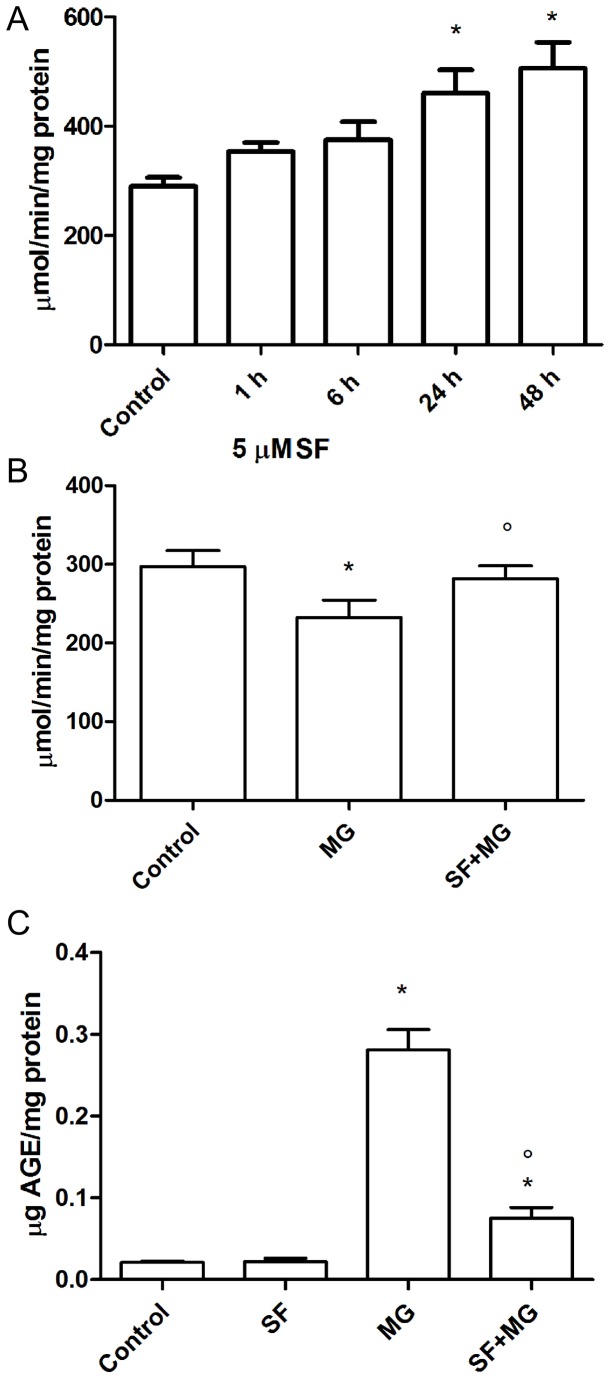
Effect of SF against MG-induced glycation. (**A**) Cardiomyocytes were treated with 5 µM SF for different time intervals (1-48 h) and GLO1 enzyme activity was measured by a spectrophotometric method. (**B**) Cardiomyocytes were treated with 5 µM SF for 24 h before addition of 1 mM MG, and GLO1 enzyme activity was measured by a spectrophotometric method. (**C**) Cardiomyocytes were treated with 5 µM SF for 24 h before addition of 1 mM MG, and AGE formation was evaluated by AGE-ELISA using a specific anti-AGE antibody. Data are presented as mean ± SD, n = 4 in each group, * p<0.05 vs Control; ° p<0.05 vs MG.

To relate GLO1 overexpression to its functional activity, cardiomyocytes were treated with 5 µM SF and GLO1 enzymatic activity was measured at different time points (1-48 h) (Figure 9A). After 1 and 6 h GLO1 activity was slightly increased but not significantly different from control cells. After 24 and 48 h SF was able to significantly increase GLO1 activity with respect to untreated cells. Figure 9B shows the effect of 1 mM MG for 24 h in the absence/presence of SF. Interestingly MG alone reduced GLO1 activity while SF pre-treatment was able to maintain GLO1 activity at level comparable to control cells. To demonstrate a direct effect of SF against MG-induced glycation, we measured AGE production in cardiomyocytes pre-treated with 5 µM SF for 24 h before exposure to 1 mM MG. Figure 9C shows that, as expected, SF did not modify AGE production with respect to control cells. On the contrary, MG strongly increased AGE production, demonstrating its ability to induce glycative stress. Interestingly, SF pre-treatment was able to significantly reduce AGE production compared to MG-treated cell.

## Discussion

SF has shown cardioprotective activity in different in vitro and in vivo models [12,38] but its multiple mechanisms of action are not yet fully understood. The goal of this study was to identify SF target proteins and mechanisms implicated in cardioprotection. A 2-DE-based proteomics approach was employed to profile the proteins affected by SF, leading to the identification of 41 distinct proteins with altered expression, which were associated with diverse biological functions. Most of these proteins, such as Elfin, Vimentin, Calreticulin, S100 calcium-binding proteins, Tropomyosin chains TPM2 and TPM4 were evidenced to be binding proteins. Twelve percent of SF responsive proteins, such as Peroxiredoxin-6, Phosphoglycerate mutase 1, Superoxide dismutase 1 and Fructose-bisphosphate aldolase were associated with catalytic activity. 

Unexpectedly, proteomic analysis did not reveal the modulation of glutathione reductase (GR), glutathione-S-transferase (GST), NAD(P)H:quinone oxidoreductase-1 (NQO1), and thioredoxin reductase (TR) although in a previous study we reported their induction by SF [12]. Regarding GR and TR, we previously observed an increase in protein abundance less than 2-fold compared to the control, so it is not surprising we did not find GR and TR differentially expressed in our proteomic study because we chose only protein spots with a fold change of at least ± 1.5-fold compared to control. On the contrary, based on the results of our previous work, we expected to identify GST and NQO1 as they were strongly up-regulated by SF. The failure of not finding them could be ascribed to the different extraction protocols that may have impacted on the yield of these specific enzymes and molecular weight/isoelectric point. Our 2-DE maps are generally well resolved at those values of molecular weight and isoelectric points but we cannot exclude that those enzymes, as well as other proteins, have undergone posttranslational modifications with important shifts towards low-resolution gel regions with overlapping, less defined and/or separated spots.

Among the 41 identified proteins, we focused our attention on 3 of these proteins because of their possible involvement in multiple cellular pathways related to cardioprotection (e.g. apoptosis, detoxification, antioxidant activity, cytoskeleton stability): MIF, CLP36, and GLO1.

MIF has emerged as a key player in cardiovascular diseases [39,40]. An up-regulation of myocardial MIF expression has been observed in surviving cardiomyocytes and macrophages in a rat model of acute myocardial ischemic injury [41]. MIF has also been reported to be released by ischemic heart tissue and to activate the cardioprotective AMP-activated protein kinase (AMPK) pathway [42]. MIF is characterized by two conserved sequence motifs that have been found to represent local catalytic centres responsible for two distinct enzymatic activities, a tautomerase/isomerase and a thiol-protein oxidoreductase activity [43]. Several studies suggest that the tautomerase/isomerase activity is related to its proinflammatory function [44], whereas its oxidoreductase activity may play a role in regulating cellular redox homeostasis [45]. Recently, Koga et al. [35] provided the first in vivo evidence supporting a role for the oxidoreductase function of MIF in mediating cardioprotection during I/R by reducing the oxidative environment of the myocardium. Moreover, it has been reported that a number of isothiocyanates, including SF, potently and irreversibly inactivate the MIF tautomerase enzymatic activity [46]. In particular, studies using neutralizing antibodies or MIF deficient animals showed that inhibition of MIF can ameliorate disease development in animal models of inflammatory bowel disease and arthritis [47,48]. Our data, for the first time, demonstrated that SF, besides its reported ability to inhibit MIF tautomerase activity, increases MIF protein level probably boosting its protective oxidoreductase activity. This result is very important because it could contribute to explain the previously demonstrated SF cardioprotective effect against oxidative stress [12,17]. Further studies will be necessary to better clarify the exact role of MIF enzymatic activity in cardioprotection.

CLP36 (CLIM1, Elfin, PDLIM1) is a member of the ALP/Enigma protein family [49] that serves as scaffolding proteins at the actin cytoskeleton. CLP36 contains a N-terminal PDZ domain and a C-terminal LIM domain and is abundantly expressed in the heart [50]. Although the physiological functions of CLP36 are not fully elucidated, several proteins have been shown to interact with cytoskeletal proteins through their PDZ domain and also with intracellular kinases through their LIM domain(s) [51]. In particular, it has been observed that CLP36 recruits the Clik1 kinase to actin cytoskeleton and this association is mediated through a bridge formed by the PDZ-LIM protein and α-actinin [52]. In a previous work we demonstrated that hypoxia is able to impair cardiomyocyte actin organization [53], so it could be hypothesized that SF up-regulation of CLP36 is involved in the maintenance of the cytoskeleton structure integrity.

GLO1 is part of the glyoxalase system that catalyses the conversion of reactive, acyclic α-oxoaldehydes into the corresponding α-hydroxyacids [13]. The main physiological GLO1 substrate is MG, and this accumulates markedly when the enzyme is inhibited in situ by cell-permeable GLO1 inhibitors and by depletion of GSH [54]. MG, a highly reactive carbonyl compound generated by carbohydrate oxidation and glycolysis, is the major precursor of protein glycation [16]. MG is more reactive than its parent sugar glucose, showing a stronger ability to cross-link with protein amino groups to form AGEs [55]. The accumulation of AGEs has been closely associated with chronic diseases caused by either or both hyperglycemia and oxidative stress [56,57]. It has been observed that MG-AGEs induced cardiac myocyte injury accompanied with temporal activation of ERK1/2, p38 MAPK and nuclear O-GlcNAcylation, indicating their potential roles in the AGE-triggered ROS generation and apoptosis [58]. Our data confirmed MG deleterious effect on cardiomyocytes, moreover, for the first time, we demonstrated that SF counteracted MG-induced apoptotic cell death, reduced ROS generation and AGE production, and induced GLO1 expression and functional activity. Likely, SF elicits a multi-target behavior against MG-induced damage: it up-regulates GLO1 enhancing the cell ability to detoxify MG, and acts as an indirect antioxidant reducing ROS produced by MG. Even if the exact role of the glyoxalase system in cardiovascular disease has not been fully elucidated, it has been observed that in hemodialysis patients, the GLO1 A419C polymorphism is associated with a significantly higher prevalence of cardiovascular disease and peripheral vascular disease risk [59]. Moreover, Wang et al. [60] demonstrated that thioredoxin activity was decreased due to post-translational glycative modification in cardiomyocytes treated with MG and blocking AGE production inhibited thioredoxin inactivation and significantly protected cardiomyocytes from I/R injury. These results suggest that any intervention able to reduce intracellular MG concentration, like GLO1 induction by SF treatment, could attenuate injury endured in myocardial I/R processes.

In conclusion, the identification of proteins with altered expression profiles at multiple treatment time points may serve to elucidate the cellular mechanisms involved in the response to SF in cardiac cells. In particular, the up-regulation of MIF, CLP36 and GLO1, which are associated with anti-apoptotic, cytoskeleton binding, and detoxifying activities, gives new insights into the understanding of SF pleiotropic behavior in counteracting cardiovascular diseases.

## Supporting Information

Figure S1 **Two-way hierarchical clustering of the 64 differentially expressed protein spots between DMSO vehicle control (CTR) and SF-treated cardiomyocytes for 1, 6, 24 and 48 h.** Pearson's dissimilarity as distance measure and Ward's method for linkage analysis were used. Log_2_ ratios are colour coded as indicated. Gene names and IDs of the identified protein spots are shown on the right (see Table S1).
(DOC)

Figure S2 **Representative MALDI-ToF spectra and mass lists for Elfin (CLP36) (spot no. 23, A), GLO1 (spot no. 32, B) and MIF (spot no. 46, C).**
(DOC)

Figure S3 **Temporal expression profiles following SF exposure of the 41 uniquely identified proteins grouped according to Gene Ontology (GO) molecular function.** Log_10_ ratio values are shown.
(DOC)

Figure S4 **Enrichment analysis of the identified proteins differentially expressed between SF-treated cardiomyocytes and DMSO vehicle control.** (**A**) Enrichment of GeneGo pathway maps. (**B**) Enrichment of GeneGo metabolic networks. (**C**) Enrichment of GeneGo process networks.
(DOC)

Figure S5 **Identification of IC50 for MG cytotoxicity.** Cardiomyocytes were treated with 0.1-5 mM MG and after 24 h cell viability was assessed by MTT assay and reported as percent cell viability in comparison to control. * p<0.05 vs Control.
(DOC)

Table S1 **SF-modified protein spots identified by MS.**
(DOC)

Table S2 **GO categories of SF responsive proteins identified by MS.**
(DOC)

Table S3 **Statistics of MetaCore network analysis of proteomic data and significant functional protein subnetworks using “analyze network” algorithm.**
(DOC)

Table S4 **Statistics of MetaCore network analysis of proteomic data and significant functional protein subnetworks using “transcription regulation” algorithm.**
(DOC)
