# Supplementary figures and images for: Novel Targets of Sulforaphane in Primary Cardiomyocytes Identified by Proteomic Analysis
Source: PLoS One. 2013 Dec 11;8(12):e83283. doi: 10.1371/journal.pone.0083283 (PMC3859650; doi:10.1371/journal.pone.0083283)

**
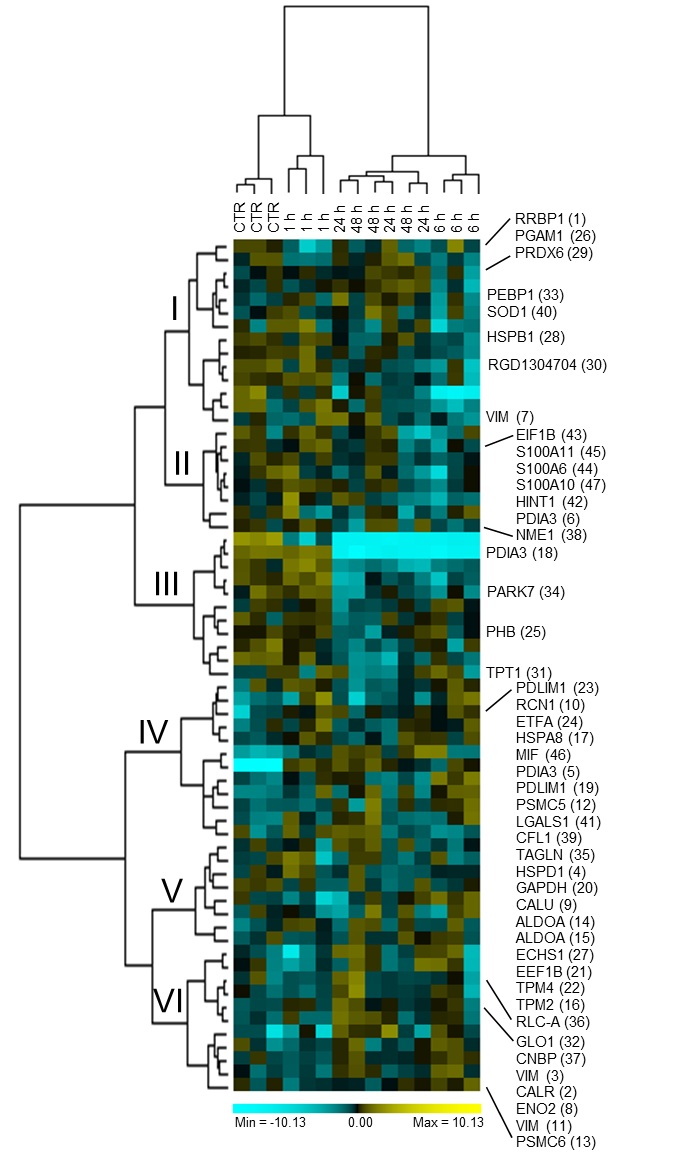
**

Supplement: Figure S1 — Two-way hierarchical clustering of the 64 differentially expressed protein spots between DMSO vehicle control (CTR) and SF-treated cardiomyocytes for 1, 6, 24 and 48 h. Pearson's dissimilarity as distance measure and Ward's method for linkage analysis were used. Log2 ratios are colour coded as indicated. Gene names and IDs of the identified protein spots are shown on the right (see Table S1). (DOC) [file pone.0083283.s001.doc]

**A)**


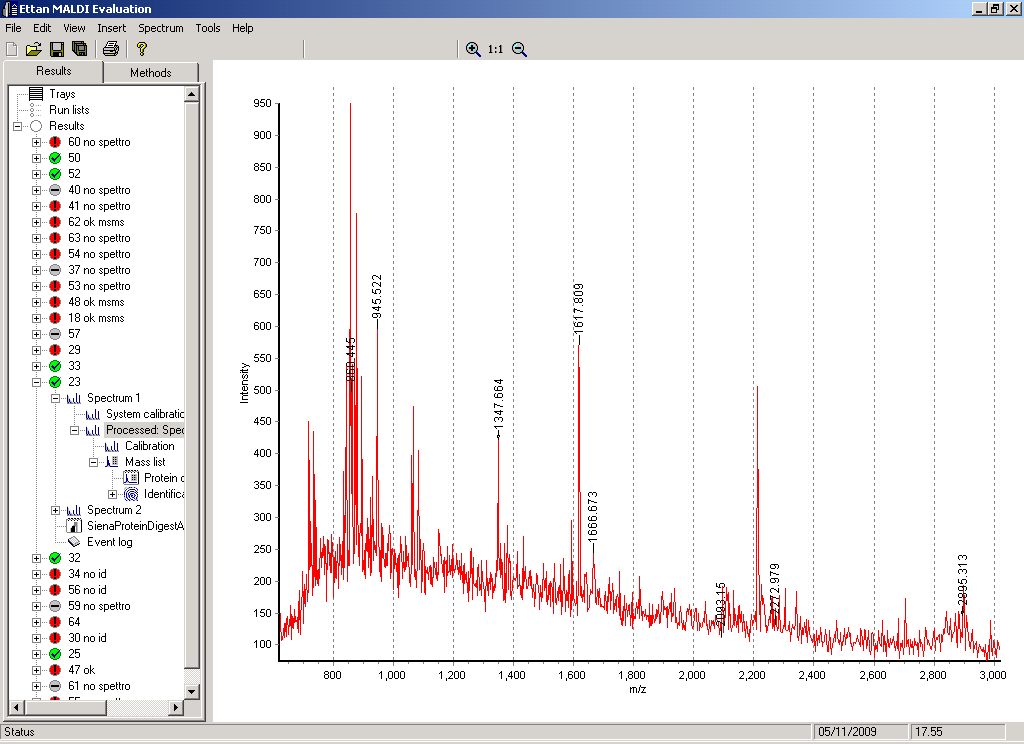


860.445

945.522

1347.664

1617.809

1666.673

2093.15

2272.979

2895.313

**B)**


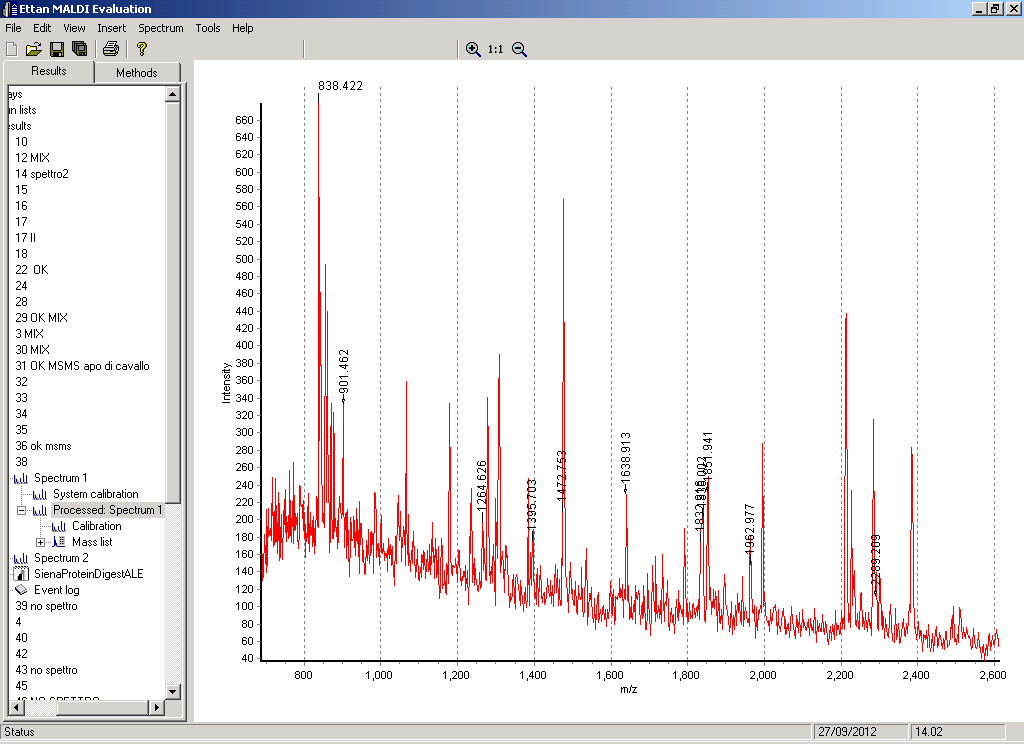


838.422

901.462

1264.626

1395.703

1472.753

1638.913

1832.916

1838.002

1851.941

1962.977

2289.209

**C)**


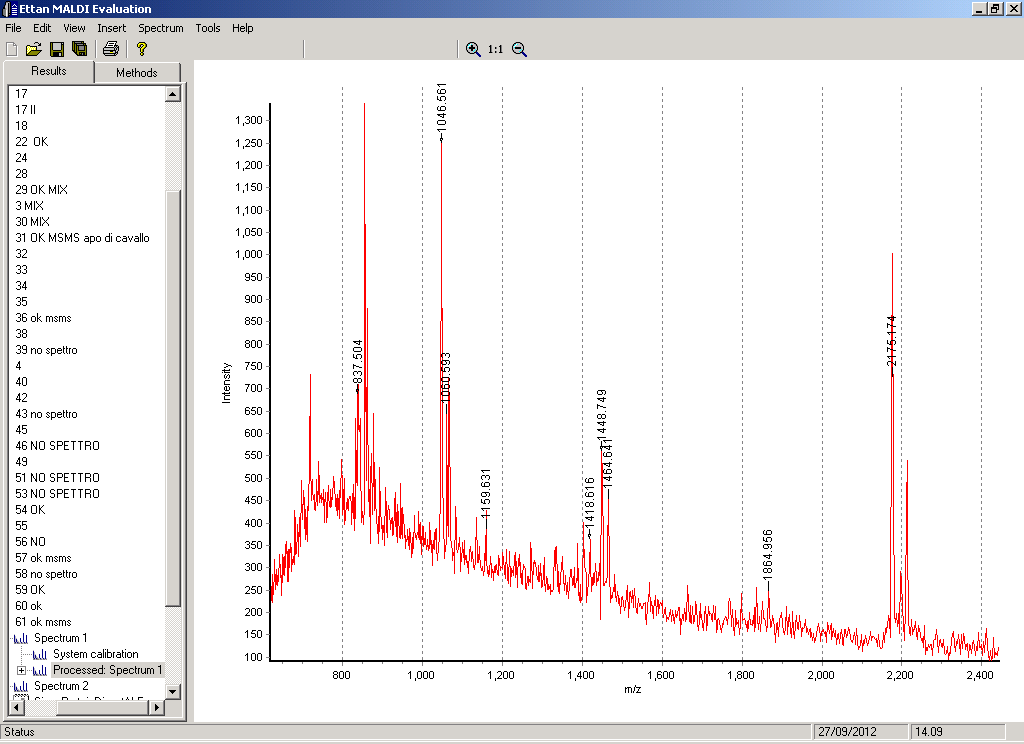


837.504

1046.561

1060.593

1159.631

1418.616

1448.749

1464.641

1864.956

2175.174

Supplement: Figure S2 — Representative MALDI-ToF spectra and mass lists for Elfin (CLP36) (spot no. 23, A), GLO1 (spot no. 32, B) and MIF (spot no. 46, C). (DOC) [file pone.0083283.s002.doc]

**
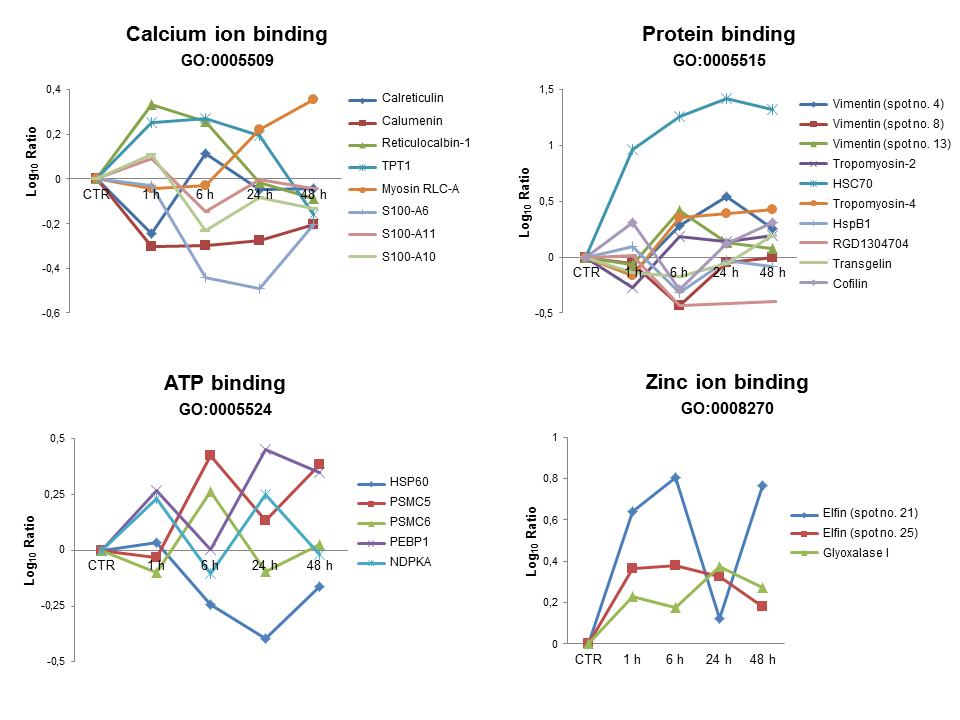
**

**
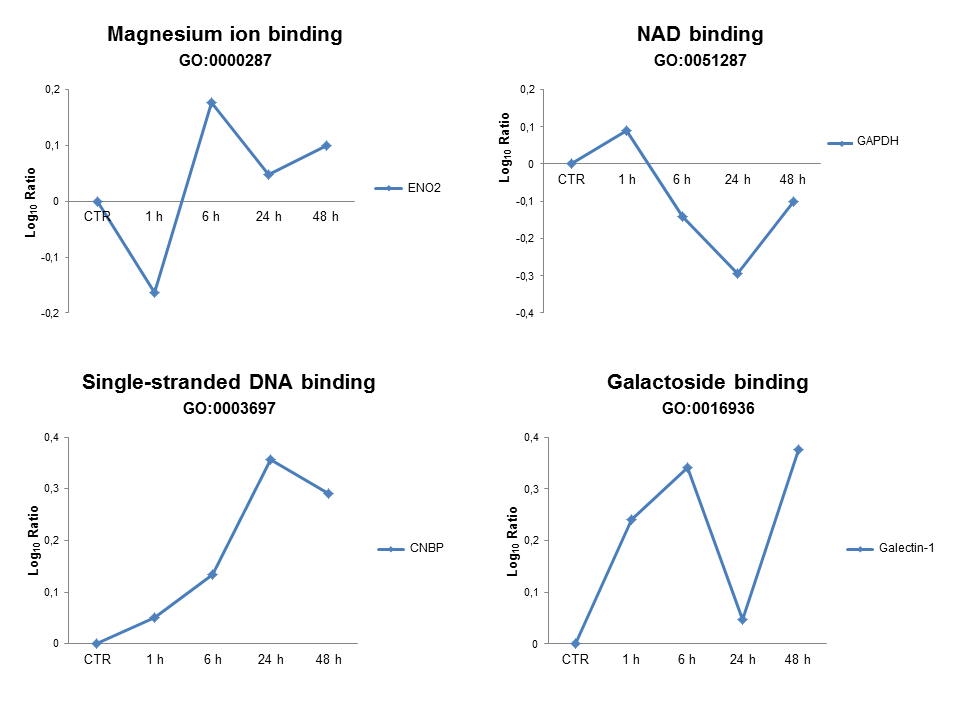
**

**
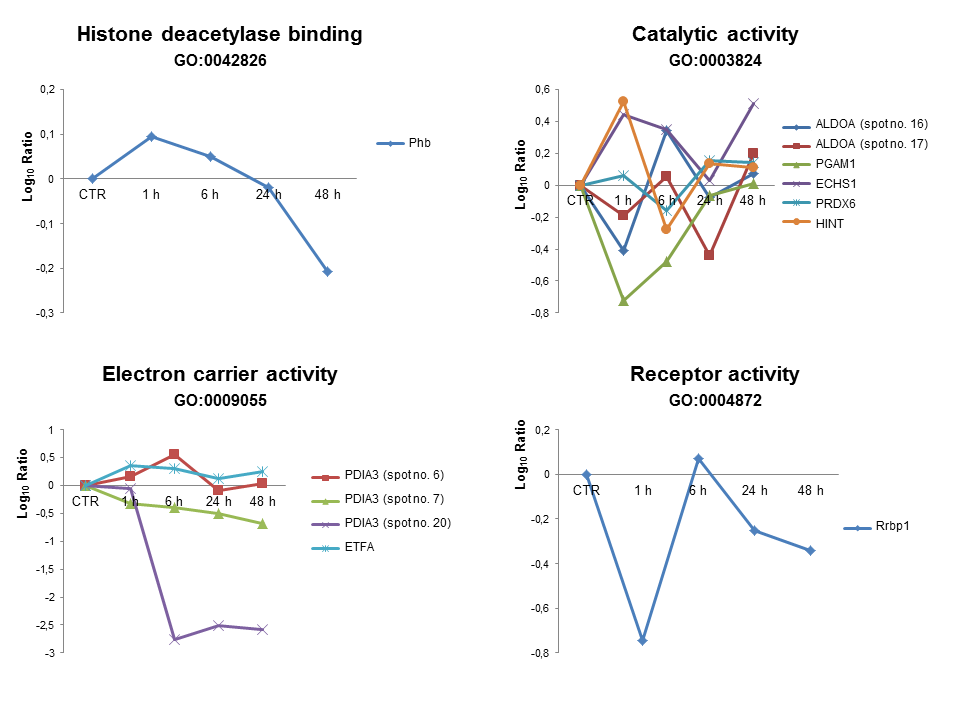
**

**
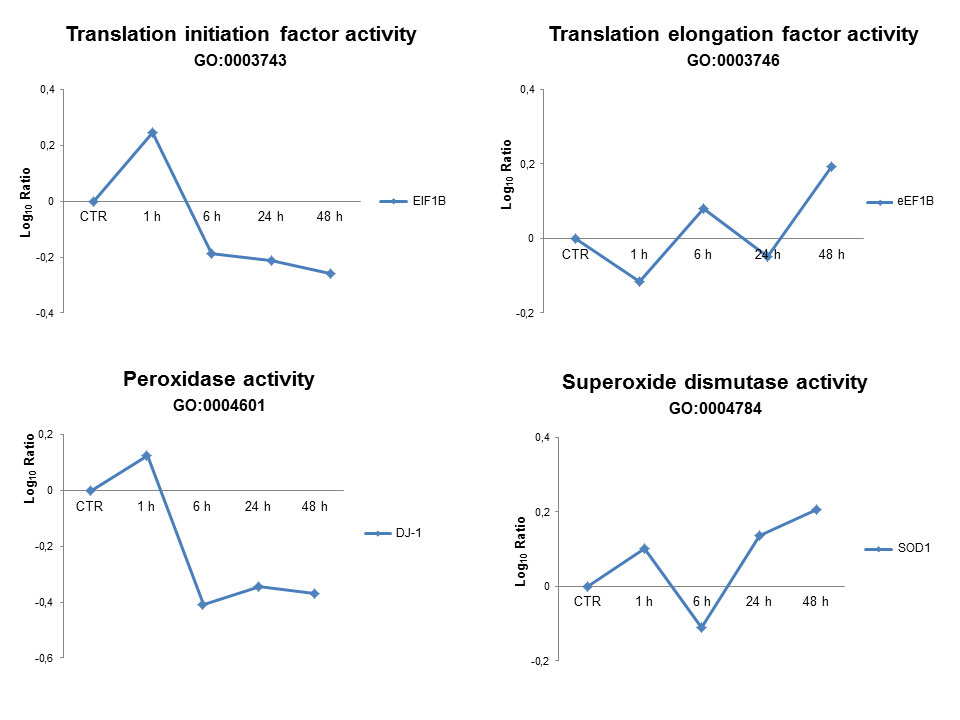
**

**
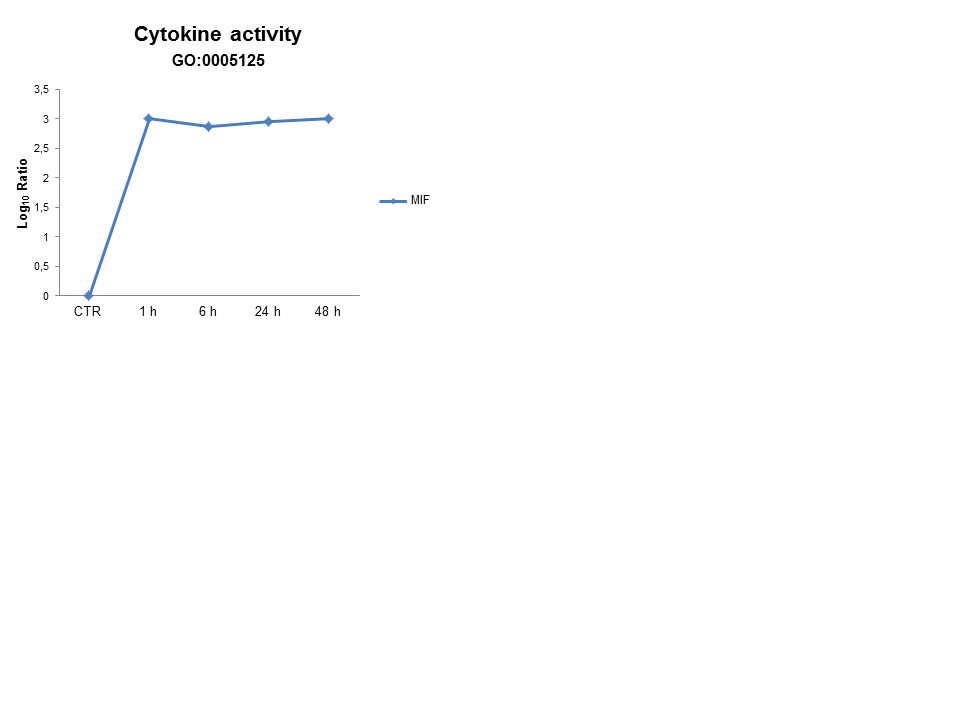
**

Supplement: Figure S3 — Temporal expression profiles following SF exposure of the 41 uniquely identified proteins grouped according to Gene Ontology (GO) molecular function. Log10 ratio values are shown. (DOC) [file pone.0083283.s003.doc]

**
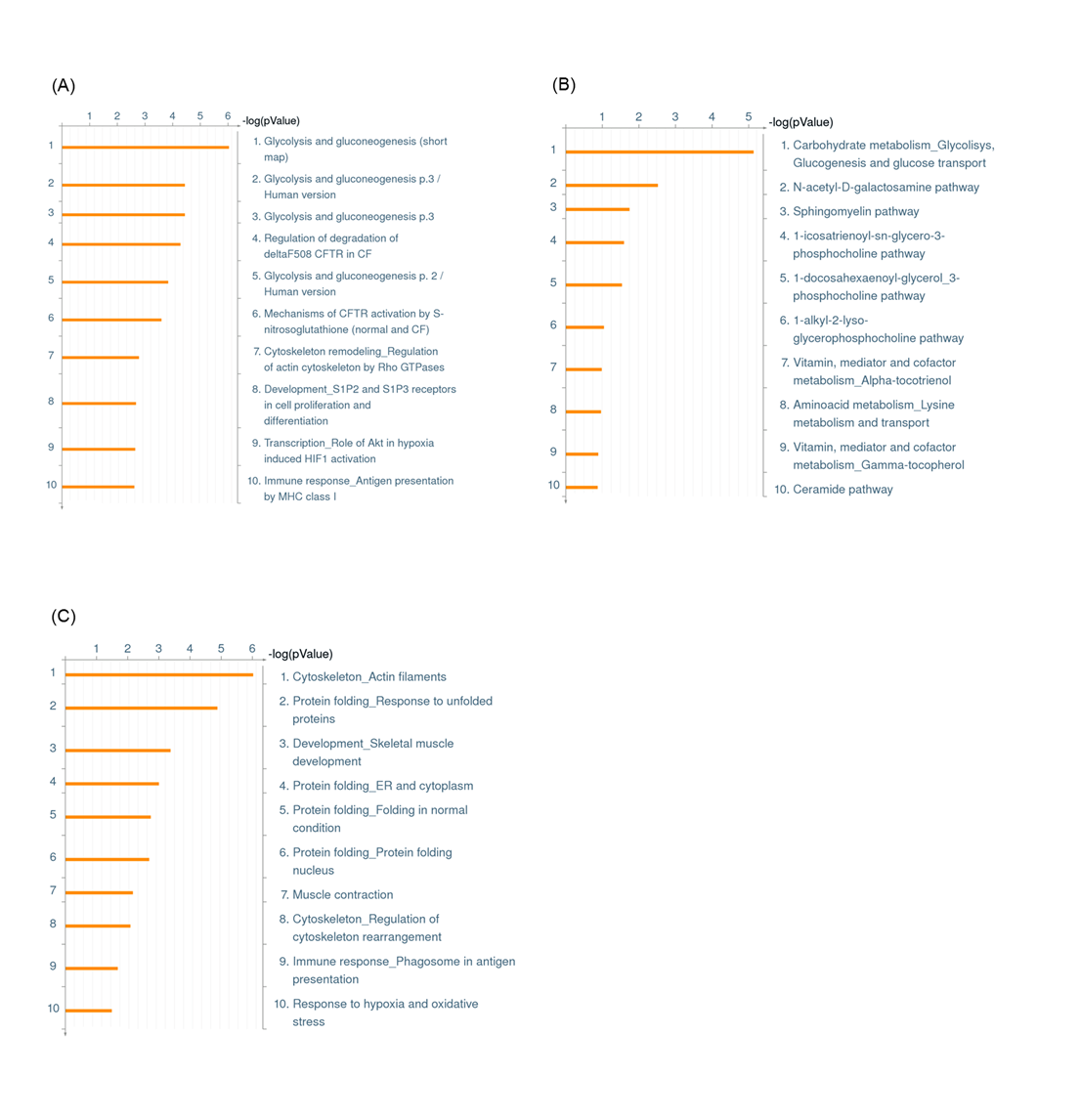
**

Supplement: Figure S4 — Enrichment analysis of the identified proteins differentially expressed between SF-treated cardiomyocytes and DMSO vehicle control. (A) Enrichment of GeneGo pathway maps. (B) Enrichment of GeneGo metabolic networks. (C) Enrichment of GeneGo process networks. (DOC) [file pone.0083283.s004.doc]
