# Supplementary material for: Novel Targets of Sulforaphane in Primary Cardiomyocytes Identified by Proteomic Analysis
Source: PLoS One. 2013 Dec 11;8(12):e83283. doi: 10.1371/journal.pone.0083283 (PMC3859650; doi:10.1371/journal.pone.0083283)
Supplement: Table S1 — SF-modified protein spots identified by MS. (DOC) [file pone.0083283.s006.doc]

| Spot ID | Gene name | Protein name | UniProt ID or NCBInr AN^§^ | Theoretical | | Experimental | | MASCOT search results | | | | | Fold ratio to control | | | |
| --- | --- | --- | --- | --- | --- | --- | --- | --- | --- | --- | --- | --- | --- | --- | --- | --- |
|  |  |  |  |  |  |  |  | Sequence coverage (%) | Score | | Matching peptides | |  |  |  |  |
|  |  |  |  | MW | pI | MW | pI |  |  |  |  |  | 1 h | 6 h | 24 h | 48 h |
| 1 | RRBP1 | Protein Rrbp1 | gi|293346337 | 143.2 | 9.0 | 77.9 | 4.8 | 16 | 169 | | 19/24 | | -5.5 | NS | -1.8 | -2.2 |
| 2 | CALR | Calreticulin | P18418 | 48.0 | 4.3 | 72.1 | 3.7 | 45 | 201 | | 15/20 | | -1.8 | NS | NS | NS |
| 3 | VIM | Vimentin | P31000 | 53.7 | 5.0 | 64.1 | 5.0 | 56 | 294 | | 27/34 | | NS | 1.9 | 3.5 | 1.8 |
| 4 | HSPD1 | 60 kDa heat shock protein | P63039 | 60.9 | 5.9 | 66.8 | 5.5 | 45 | 230 | | 21/28 | | NS | -1.7 | -2.5 | -1.5 |
| 5 | PDIA3 | Protein disulfide-isomerase A3 | P11598 | 56.6 | 5.9 | 66.5 | 5.8 | 46 | 278 | | 21/28 | | NS | 3.6 | NS | NS |
| 6 | PDIA3 | Protein disulfide-isomerase A3 | P11598 | 56.6 | 5.9 | 65.9 | 5.9 | 42 | 234 | | 21/33 | | -2.2 | -2.5 | -3.2 | -4.8 |
| 7 | VIM | Vimentin | P31000 | 53.7 | 5.0 | 58.5 | 4.9 | 61 | 322 | | 30/43 | | NS | -2.7 | NS | NS |
| 8 | ENO2 | Gamma-enolase | P07323 | 47.1 | 5.0 | 54.4 | 4.9 | 30 | 108 | | 10/19 | | -1.5 | 1.5 | NS | NS |
| 9 | CALU | Calumenin | O35783 | 37.1 | 4.5 | 55.3 | 3.8 | 35 | 122 | | 10/21 | | -2.0 | -2.0 | -1.9 | -1.6 |
| 10 | RCN1 | Reticulocalbin-1 | gi|157819753 | 38.1 | 4.7 | 46.3 | 4.0 | 28 | 114 | | 9/14 | | 2.2 | 1.8 | NS | NS |
| 11 | VIM | Vimentin | P31000 | 53.7 | 5.0 | 47.0 | 4.5 | 56 | 301 | | 28/38 | | NS | 2.6 | NS | NS |
| 12 | PSMC5 | 26S protease regulatory subunit 8 | P62198 | 45.6 | 7.1 | 46.5 | 7.6 | 25 | 127 | | 10/13 | | NS | 2.6 | 1.5 | 2.4 |
| 13 | PSMC6 | 26S protease regulatory subunit S10B | gi|213972598 | 44.2 | 7.1 | 44.7 | 7.7 | 30 | 183 | | 14/14 | | NS | 1.8 | NS | NS |
| 14 | ALDOA | Fructose-bisphosphate aldolase A | P05065 | 39.4 | 8.3 | 42.7 | 8.6 | 36 | 139 | | 12/15 | | -2.6 | 2.2 | NS | NS |
| 15 | ALDOA | Fructose-bisphosphate aldolase A | P05065 | 39.4 | 8.3 | 42.7 | 8.8 | 40 | 175 | | 14/17 | | -1.5 | NS | -2.7 | 1.6 |
| 16 | TPM2 | Tropomyosin beta chain | P58775 | 32.8 | 4.7 | 41.9 | 4.3 | 27 | 103 | | 10/21 | | -1.9 | 1.5 | 1.5 | 1.6 |
| 17 | HSPA8 | Heat shock cognate 71 kDa protein | P63018 | 70.9 | 5.4 | 38.3 | 4.5 | 19 | 113 | | 9/12 | | 9.1 | 18.1 | 26.4 | 20.7 |
| 18 | PDIA3 | Protein disulfide-isomerase A3 | P11598 | 56.6 | 5.9 | 38.8 | 6.7 | 17 | 110 | | 9/10 | | NS | -571.3 | -320.7 | -380.3 |
| 19 | PDLIM1 | PDZ and LIM domain protein 1 (Elfin) | P52944 | 35.6 | 6.8 | 38.4 | 7.0 | 32 | 115 | | 10/15 | | 4.4 | 6.4 | NS | 5.9 |
| 20 | GAPDH | Glyceraldehyde-3-phosphate dehydrogenase | P04797 | 35.8 | 8.1 | 37.9 | 8.7 | 36 | 121 | | 9/13 | | NS | NS | -2.0 | NS |
| 21 | EEF1B | Elongation factor 1-beta | gi|157818179 | 24.7 | 4.5 | 33.0 | 4.0 | 37 | 131 | | 7/8 | | NS | NS | NS | 1.6 |
| 22 | TPM4 | Tropomyosin alpha-4 chain | P09495 | 28.5 | 4.7 | 33.9 | 4.3 | 39 | 130 | | 10/20 | | -1.5 | 2.3 | 2.4 | 2.7 |
| 23 | PDLIM1 | PDZ and LIM domain protein 1 (Elfin) | P52944 | 35.6 | 6.8 | 36.4 | 7.1 | 30 | 135 | | 11/15 | | 2.3 | 2.4 | 2.1 | 1.5 |
| 24 | ETFA | Electron transfer flavoprotein subunit alpha | P13803 | 35.0 | 8.6 | 32.9 | 7.5 | 28 | 101 | | 7/11 | | 2.2 | 2.0 | 1.5 | 1.7 |
| 25 | PHB | Prohibitin | P67779 | 29.8 | 5.6 | 31.4 | 5.6 | 32 | 107 | | 7/11 | | NS | NS | NS | -1.6 |
| 26 | PGAM1 | Phosphoglycerate mutase 1 | P25113 | 28.8 | 6.7 | 29.0 | 6.6 | 30 | 116 | | 7/9 | | -5.3 | -3.0 | NS | NS |
| 27 | ECHS1 | Enoyl-CoA hydratase | P14604 | 31.5 | 8.4 | 28.0 | 7.1 | 32 | 116 | | 9/18 | | 2.8 | 2.2 | NS | 3.2 |
| 28 | HSPB1 | Heat shock protein beta-1 | P42930 | 22.9 | 6.1 | 28.0 | 5.4 | 36 | 141 | | 8/10 | | NS | -2.1 | NS | NS |
| 29 | PRDX6 | Peroxiredoxin-6 | O35244 | 24.8 | 5.6 | 26.8 | 6.0 | 53 | 125 | | 8/14 | | NS | -1.5 | 1.5 | 1.5 |
| 30 | RGD 1304704 | Similar to hypothetical protein CGI-99, isoform CRA_c | gi|149031197 | 28.2 | 6.4 | 26.7 | 6.7 | 39 | 142 | | 9/11 | | NS | -2.7 | -2.6 | -2.5 |
| 31 | TPT1 | Translationally-controlled tumor protein | P63029 | 19.5 | 4.8 | 25.0 | 4.3 | 37 | 103 | | 8/10 | | 1.8 | 1.9 | 1.6 | NS |
| 32 | GLO1 | Lactoylglutathione lyase | Q6P7Q4 | 20.8 | 5.1 | 25.1 | 4.7 | 41 | 113 | | 7/11 | | 1.7 | 1.5 | 2.4 | 1.9 |
| 33 | PEBP1 | Phosphatidylethanolamine-binding protein 1 | P31044 | 20.8 | 5.5 | 23.4 | 5.2 | 67 | 154 | | 8/10 | | 1.8 | NS | 2.8 | 2.2 |
| 34 | PARK7 | Protein DJ-1 | O88767 | 20.0 | 6.3 | 22.5 | 6.3 | 48 | 113 | | 8/9 | | NS | -2.6 | -2.2 | -2.3 |
| 35 | TAGLN | Transgelin | P31232 | 22.6 | 8.9 | 23.6 | 8.8 | 65 | 187 | | 19/27 | | NS | -1.5 | NS | 1.5 |
| 36 | RLC-A | Myosin regulatory light chain RLC-A | P13832 | 19.9 | 4.7 | 20.4 | 4.4 | 41 | 91 | | 7/11 | | NS | NS | 1.7 | 2.3 |
| 37 | CNBP | Cellular nucleic acid-binding protein | P62634 | 19.5 | 8.0 | 17.7 | 4.9 | 44 | 114 | | 7/12 | | NS | NS | 2.3 | 1.9 |
| 38 | NME1 | Nucleoside diphosphate kinase A | Q05982 | 17.2 | 5.9 | 17.6 | 6.0 | 48 | 100 | | 7/11 | | 1.7 | NS | 1.8 | NS |
| 39 | CFL1 | Cofilin-1 | P45592 | 18.5 | 8.2 | 17.7 | 8.6 | 46 | 116 | | 8/17 | | 2.0 | -1.9 | NS | 2.0 |
| 40 | SOD1 | Superoxide dismutase [Cu-Zn] | P07632 | 15.9 | 5.9 | 16.7 | 6.1 | 47 | 133 | | 7/10 | | NS | NS | NS | 1.6 |
| 41 | LGALS1 | Galectin-1 | P11762 | 14.9 | 5.1 | 14.7 | 4.5 | 43 | 118 | | 6/8 | | 1.7 | 2.2 | NS | 2.4 |
| 42 | HINT1 | Histidine triad nucleotide-binding protein 1 | P62959 | 13.8 | 6.4 | 14.3 | 6.5 | 55 | 86 | | 5/10 | | 3.4 | -1.9 | NS | NS |
| 43 | EIF1B | Eukaryotic translation initiation factor 1b | gi|149054210 | 12.8 | 6.8 | 13.3 | 7.2 | 48 | 70 | | 4/8 | | 1.8 | -1.5 | -1.6 | -1.8 |
|  |  |  |  |  |  |  |  | TLTTVQGIADDYDK | | | | |  |  |  |  |
| 44 | S100A6 | Protein S100-A6 | P05964 | 10.0 | 5.3 | 11.2 | 4.8 | 52 | | 68 | | 5/12 | NS | -2.7 | -3.1 | -1.6 |
|  |  |  |  |  |  |  |  | LMDDLDR  LmDDLDR^¥^  LQDAEIAR | | | | |  |  |  |  |
| 45 | S100A11 | Protein S100-A11 | Q6B345 | 11.1 | 5.6 | 12.0 | 5.9 | 51 | | 89 | | 6/10 | NS | -1.5 | NS | NS |
|  |  |  |  |  |  |  |  | CIESLIAVFQK  TEFLSFMNTELAAFTK  TEFLSFmNTELAAFTK^¥^ | | | | |  |  |  |  |
| 46 | MIF | Macrophage migration inhibitory factor | P30904 | 12.5 | 6.8 | 12.2 | 6.6 | 24 | | 68 | | 4/9 | 1032.1 | 741.0 | 906.2 | 1028.0 |
|  |  |  |  |  |  |  |  | LLCGLLSDR | | | | |  |  |  |  |
| 47 | S100A10 | Protein S100-A10 | P05943 | 11.1 | 6.3 | 11.3 | 6.8 | 33 | | 69 | | 4/6 | NS | -1.7 | NS | NS |

^§^UniProtKB database Identifier or NCBInr database accession number.

^¥^MS/MS sequenced peptide in which the Met residue is oxidized (m).

NS, not statistically significant (Student’s *t*-test).
