# Supplementary material for: Novel Targets of Sulforaphane in Primary Cardiomyocytes Identified by Proteomic Analysis
Source: PLoS One. 2013 Dec 11;8(12):e83283. doi: 10.1371/journal.pone.0083283 (PMC3859650; doi:10.1371/journal.pone.0083283)
Supplement: Table S2 — GO categories of SF responsive proteins identified by MS. (DOC) [file pone.0083283.s007.doc]

| Protein name | Biological process | Cellular component | Molecular function |
| --- | --- | --- | --- |
| Protein Rrbp1 | NA | ER | Receptor activity |
| Calreticulin | Protein folding | ER | Calcium ion binding |
| Vimentin | Intermediate filament organization | Cytoplasm | Protein binding |
| HSP60 | Protein folding | Mitochondrion | ATP binding |
| PDIA3 | Cell redox homeostasis | ER | Electron carrier activity |
| ENO2 | Glycolysis | Cytoplasm | Magnesium ion binding |
| Calumenin | Negative regulation of catalytic activity | ER | Calcium ion binding |
| Reticulocalbin-1 | Camera-type eye development | ER | Calcium ion binding |
| PSMC5 | Protein catabolic process | Cytoplasm | ATP binding |
| PSMC6 | Protein catabolic process | Cytoplasm | ATP binding |
| ALDOA | Glycolysis | Cytoplasm | Catalytic activity |
| Tropomyosin-2 | Regulation of ATPase activity | Cytoplasm | Actin binding |
| HSC70 | Protein folding | Cytoplasm | Protein binding |
| Elfin | Transcription | Cytoplasm | Zinc ion binding |
| GAPDH | Glycolysis | Cytoplasm | NAD binding |
| eEF1B | Translation | Cytoplasm | Translation elongation factor activity |
| Tropomyosin-4 | Muscle contraction | Cytoplasm | Actin binding |
| ETFA | Electron transport chain | Mitochondrion | Electron carrier activity |
| Phb | DNA replication | Mitochondrion | Histone deacetylase binding |
| PGAM1 | Glycolysis | Nucleus | Catalytic activity |
| ECHS1 | Lipid metabolic process | Mitochondrion | Catalytic activity |
| HspB1 | Anti-apoptosis | Cytoplasm | Protein binding |
| PRDX6 | Oxidation-reduction process | Cytoplasm | Catalytic activity |
| Protein RGD1304704 | Transcription | Nucleus | Protein binding |
| TPT1 | Anti-apoptosis | Cytoplasm | Calcium ion binding |
| Glyoxalase I | Anti-apoptosis | Cytoplasm | Zinc ion binding |
| PEBP1 | Positive regulation of acetylcholine metabolic process | Cytoplasm | ATP binding |
| DJ-1 | Oxidation-reduction process | Cytoplasm | Peroxidase activity |
| Transgelin | Cytoskeleton organization | Cytoplasm | Actin binding |
| Myosin RLC-A | Regulation of cell shape | Myosin II complex | Calcium ion binding |
| CNBP | Transcription | Cytoplasm | Single-stranded DNA binding |
| NDPKA | Nucleotide metabolic process | Cytoplasm | ATP binding |
| Cofilin | Cytoskeleton organization | Cytoplasm | Actin binding |
| SOD1 | Oxidation-reduction process | Cytoplasm | Superoxide dismutase activity |
| Galectin-1 | Regulation of cell-substrate adhesion | Extracellular space | Galactoside binding |
| HINT | Intracellular protein transport | Cytoplasm | Catalytic activity |
| EIF1B | Translation | Cytoplasm | Translation initiation factor activity |
| S100-A6 | Transmembrane transport | Plasma membrane | Calcium ion binding |
| S100-A11 | NA | Cytoplasm | Calcium ion binding |
| MIF | Inflammatory response | Extracellular space | Cytokine activity |
| S100-A10 | Regulation of cell growth | ER | Calcium ion binding |

NA, not available.

ER, endoplasmic reticulum.
