# Supplementary material for: Novel Targets of Sulforaphane in Primary Cardiomyocytes Identified by Proteomic Analysis
Source: PLoS One. 2013 Dec 11;8(12):e83283. doi: 10.1371/journal.pone.0083283 (PMC3859650; doi:10.1371/journal.pone.0083283)
Supplement: Table S3 — Statistics of MetaCore network analysis of proteomic data and significant functional protein subnetworks using “analyze network” algorithm. (DOC) [file pone.0083283.s008.doc]

| # | Network | GO processes | Seed nodes | p-Value | zScore | gScore |
| --- | --- | --- | --- | --- | --- | --- |
| 1 | DJ-1, HSP60, NF-kB, TAK1(MAP3K7), TRAF6 | positive regulation of immune system process (85.7%; 3.255e-55), MyD88-dependent toll-like receptor signaling pathway (57.1%; 1.111e-54), toll-like receptor signaling pathway (59.2%; 1.526e-54), positive regulation of innate immune response (63.3%; 1.727e-53), positive regulation of defense response (69.4%; 1.769e-53) | 2 | 4.740E-04 | 11.04 | 1901.04 |
| 2 | TPT1, SOD1, HSP27, RKIP, Glyoxalase I | toll-like receptor 4 signaling pathway (34.7%; 4.316e-28), positive regulation of defense response (42.9%; 3.923e-27), toll-like receptor signaling pathway (34.7%; 5.692e-27), MyD88-independent toll-like receptor signaling pathway (32.7%; 8.218e-27), toll-like receptor 1 signaling pathway (32.7%; 9.868e-27) | 11 | 5.130E-26 | 61.54 | 61.54 |
| 3 | NDPK A, MIF, Transgelin, ALDOA, MYOD | muscle system process (51.0%; 1.643e-33), muscle contraction (49.0%; 2.207e-33), actin-myosin filament sliding (28.6%; 1.013e-26), actin filament-based movement (30.6%; 7.883e-26), actin-mediated cell contraction (28.6%; 1.030e-25) | 8 | 5.400E-18 | 45.16 | 45.16 |
| 4 | Calcyclin, Calumenin, G3P2, HSP60, PSMC6 | MyD88-dependent toll-like receptor signaling pathway (41.7%; 2.327e-35), TRIF-dependent toll-like receptor signaling pathway (39.6%; 2.992e-34), toll-like receptor 3 signaling pathway (39.6%; 4.810e-34), MyD88-independent toll-like receptor signaling pathway (39.6%; 1.201e-33), toll-like receptor signaling pathway (41.7%; 1.494e-33) | 7 | 2.300E-15 | 39.1 | 39.1 |
| 5 | Calreticulin, HSP60, PDIA3, CNBP, STAT3 | positive regulation of immune response (45.7%; 4.651e-23), positive regulation of immune system process (50.0%; 3.919e-22), positive regulation of T cell mediated immunity (26.1%; 1.353e-21), positive regulation of T cell mediated cytotoxicity (23.9%; 4.502e-21), positive regulation of adaptive immune response based on somatic recombination of immune receptors built from immunoglobulin superfamily domains (28.3%; 1.636e-20) | 4 | 2.890E-08 | 22.49 | 22.49 |
| 6 | Galectin-1, RelA (p65 NF-kB subunit), PRKD2, MYH9, HSPA1B | regulation of cellular amino acid metabolic process (38.0%; 1.117e-36), negative regulation of ubiquitin-protein ligase activity involved in mitotic cell cycle (38.0%; 4.531e-35), signal transduction involved in mitotic cell cycle G1/S transition DNA damage checkpoint (38.0%; 7.866e-35), signal transduction involved in mitotic cell cycle checkpoint (38.0%; 7.866e-35), DNA damage response, signal transduction by p53 class mediator resulting in cell cycle arrest (38.0%; 7.866e-35) | 4 | 3.140E-08 | 22.26 | 22.26 |
| 7 | S100A10, CLIM1, HINT, ATF-2, Bcl-10 | positive regulation of defense response (58.3%; 6.753e-41), MyD88-independent toll-like receptor signaling pathway (45.8%; 1.069e-40), toll-like receptor signaling pathway (47.9%; 2.566e-40), toll-like receptor 4 signaling pathway (45.8%; 1.629e-39), pattern recognition receptor signaling pathway (47.9%; 4.822e-39) | 3 | 4.560E-06 | 16.65 | 16.65 |
| 8 | Vimentin, HSP27, JNK1(MAPK8), c-Rel (NF-kB subunit), PKC-delta | toll-like receptor 4 signaling pathway (47.6%; 2.136e-36), toll-like receptor signaling pathway (47.6%; 4.707e-35), MyD88-independent toll-like receptor signaling pathway (45.2%; 4.739e-35), pattern recognition receptor signaling pathway (47.6%; 5.828e-34), innate immune response-activating signal transduction (47.6%; 1.261e-33) | 2 | 3.840E-04 | 11.66 | 11.66 |
| 9 | Reticulocalbin 1, PKC, p38alpha (MAPK14), IRAK1, PKC-lambda/iota | immune response-activating signal transduction (74.3%; 4.911e-43), immune response-regulating signaling pathway (74.3%; 3.952e-42), activation of immune response (74.3%; 8.762e-40), positive regulation of immune response (77.1%; 1.949e-38), toll-like receptor 4 signaling pathway (54.3%; 4.452e-36) | 1 | 3.130E-02 | 5.43 | 5.43 |

Gene Ontology (GO) explains the functional processes associated with built network.

zScore indicates association among the functional subnetworks of the differentially expressed proteins from 2-DE analysis.

gScore modifies the zScore based on the number of canonical pathways used to build the network.
