# Supplementary material for: Novel Targets of Sulforaphane in Primary Cardiomyocytes Identified by Proteomic Analysis
Source: PLoS One. 2013 Dec 11;8(12):e83283. doi: 10.1371/journal.pone.0083283 (PMC3859650; doi:10.1371/journal.pone.0083283)
Supplement: Table S4 — Statistics of MetaCore network analysis of proteomic data and significant functional protein subnetworks using “transcription regulation” algorithm. (DOC) [file pone.0083283.s009.doc]

| # | Network | GO processes | Seed nodes | p-Value | zScore |
| --- | --- | --- | --- | --- | --- |
| 1 | SP1 | response to drug (36.0%; 3.823e-08), response to inorganic substance (36.0%; 3.918e-08), response to heat (20.0%; 1.815e-07), response to steroid hormone stimulus (32.0%; 6.410e-07), response to hydrogen peroxide (20.0%; 7.352e-07) | 24 | 3.560E-80 | 190.26 |
| 2 | c-Myc | gluconeogenesis (22.2%; 5.262e-08), hexose biosynthetic process (22.2%; 7.966e-08), glycolysis (22.2%; 1.002e-07), monosaccharide biosynthetic process (22.2%; 1.079e-07), glucose catabolic process (22.2%; 2.522e-07) | 17 | 1.180E-55 | 158.82 |
| 3 | p53 | response to unfolded protein (23.5%; 5.590e-06), response to topologically incorrect protein (23.5%; 7.285e-06), negative regulation of cellular process (64.7%; 7.649e-06), response to inorganic substance (35.3%; 9.020e-06), regulation of apoptotic process (47.1%; 1.327e-05) | 16 | 3.030E-52 | 153.8 |
| 4 | AP-1 | negative regulation of apoptotic process (46.7%; 5.487e-07), negative regulation of programmed cell death (46.7%; 5.847e-07), negative regulation of cell death (46.7%; 7.729e-07), anti-apoptosis (33.3%; 1.729e-06), response to estrogen stimulus (33.3%; 2.066e-06) | 14 | 1.720E-45 | 143.27 |
| 5 | GCR-alpha | regulation of apoptotic process (61.5%; 9.241e-07), regulation of programmed cell death (61.5%; 9.805e-07), regulation of cell death (61.5%; 1.254e-06), response to inorganic substance (46.2%; 1.394e-06), response to hydrogen peroxide (30.8%; 1.551e-06) | 12 | 8.200E-39 | 131.91 |
| 6 | Androgen receptor | protein stabilization (33.3%; 1.578e-07), regulation of protein stability (33.3%; 6.785e-07), response to inorganic substance (50.0%; 7.713e-07), posttranscriptional regulation of gene expression (41.7%; 2.875e-06), regulation of intracellular steroid hormone receptor signaling pathway (25.0%; 5.655e-06) | 11 | 1.670E-35 | 125.85 |
| 7 | CREB1 | response to drug (70.0%; 3.338e-09), response to heat (40.0%; 1.497e-07), response to inorganic substance (60.0%; 1.851e-07), response to oxidative stress (50.0%; 3.955e-07), response to hydrogen peroxide (40.0%; 4.631e-07) | 9 | 6.120E-29 | 112.79 |
| 8 | HSF1 | response to stress (100.0%; 7.205e-08), response to unfolded protein (44.4%; 3.095e-07), response to topologically incorrect protein (44.4%; 4.046e-07), response to chemical stimulus (100.0%; 7.657e-07), response to inorganic substance (55.6%; 3.545e-06) | 8 | 1.100E-25 | 105.68 |
| 9 | ESR1 (nuclear) | response to chemical stimulus (100.0%; 7.657e-07), muscle cell homeostasis (22.2%; 4.007e-05), cellular response to chemical stimulus (66.7%; 6.442e-05), secretion (44.4%; 8.480e-05), gluconeogenesis (22.2%; 1.562e-04) | 8 | 1.100E-25 | 105.68 |
| 10 | YY1 | spermatogenesis (50.0%; 3.000e-05), male gamete generation (50.0%; 3.000e-05), gamete generation (50.0%; 7.799e-05), negative regulation of steroid metabolic process (25.0%; 7.924e-05), hydrogen peroxide metabolic process (25.0%; 1.269e-04) | 7 | 1.880E-22 | 98.08 |
| 11 | C/EBPbeta | cellular response to chemical stimulus (75.0%; 2.347e-05), regulation of cell differentiation (62.5%; 4.607e-05), negative regulation of neuron projection development (25.0%; 1.491e-04), regulation of developmental process (62.5%; 2.348e-04), peptide antigen assembly with MHC class I protein complex (12.5%; 3.601e-04) | 7 | 1.880E-22 | 98.08 |
| 12 | c-Jun | response to hydrogen peroxide (37.5%; 1.818e-05), cellular response to chemical stimulus (75.0%; 2.347e-05), response to reactive oxygen species (37.5%; 4.566e-05), response to inorganic substance (50.0%; 6.285e-05), intermediate filament cytoskeleton organization (25.0%; 7.104e-05) | 7 | 1.880E-22 | 98.08 |
| 13 | EGR1 | response to drug (75.0%; 2.562e-08), cellular response to reactive oxygen species (37.5%; 3.916e-06), positive regulation of cell proliferation (62.5%; 6.440e-06), cellular response to oxidative stress (37.5%; 1.520e-05), response to hydrogen peroxide (37.5%; 1.818e-05) | 7 | 1.880E-22 | 98.08 |
| 14 | Elk-1 | isotype switching to IgG isotypes (12.5%; 3.601e-04), detection of misfolded protein (12.5%; 3.601e-04), B cell cytokine production (12.5%; 3.601e-04), regulation of immune system process (50.0%; 5.053e-04), MyD88-dependent toll-like receptor signaling pathway (25.0%; 5.517e-04) | 7 | 1.880E-22 | 98.08 |
| 15 | HNF4-alpha | regulation of cell projection organization (50.0%; 6.253e-06), regulation of developmental process (75.0%; 1.107e-05), carbohydrate homeostasis (37.5%; 2.396e-05), glucose homeostasis (37.5%; 2.396e-05), response to glucose stimulus (37.5%; 3.085e-05) | 7 | 1.880E-22 | 98.08 |
| 16 | E2F1 | reproductive process (62.5%; 1.245e-04), reproduction (62.5%; 1.287e-04), sexual reproduction (50.0%; 1.292e-04), response to estrogen stimulus (37.5%; 1.830e-04), positive regulation of cell proliferation (50.0%; 1.873e-04) | 7 | 1.880E-22 | 98.08 |
| 17 | C/EBPalpha | cellular response to lithium ion (28.6%; 2.755e-05), response to lithium ion (28.6%; 1.443e-04), peptide antigen assembly with MHC protein complex (14.3%; 3.151e-04), peptide antigen assembly with MHC class I protein complex (14.3%; 3.151e-04), positive regulation of erythrocyte aggregation (14.3%; 3.151e-04) | 6 | 3.070E-19 | 89.87 |
| 18 | VDR | regulation of apoptotic process (85.7%; 1.180e-06), regulation of programmed cell death (85.7%; 1.235e-06), regulation of cell death (85.7%; 1.495e-06), cellular homeostasis (71.4%; 3.543e-06), antigen processing and presentation of exogenous peptide antigen via MHC class I, TAP-dependent (42.9%; 3.963e-06) | 6 | 3.070E-19 | 89.87 |
| 19 | HIF1A | regulation of neurogenesis (71.4%; 2.653e-07), regulation of nervous system development (71.4%; 4.254e-07), response to steroid hormone stimulus (71.4%; 6.185e-07), regulation of cell development (71.4%; 6.681e-07), regulation of neuron projection development (57.1%; 1.557e-06) | 6 | 3.070E-19 | 89.87 |
| 20 | AP-4 | negative regulation of apoptotic process (71.4%; 1.541e-06), negative regulation of programmed cell death (71.4%; 1.614e-06), negative regulation of cell death (71.4%; 1.981e-06), negative regulation of cell cycle arrest (28.6%; 3.824e-06), response to heat (42.9%; 4.905e-06) | 6 | 3.070E-19 | 89.87 |
| 21 | TBP | multi-organism process (71.4%; 4.240e-05), intermediate filament cytoskeleton organization (28.6%; 5.333e-05), intermediate filament-based process (28.6%; 6.936e-05), hydrogen peroxide metabolic process (28.6%; 9.532e-05), anti-apoptosis (42.9%; 1.039e-04) | 6 | 3.070E-19 | 89.87 |
| 22 | SRF | actin filament-based process (66.7%; 3.092e-06), cellular component movement (66.7%; 1.061e-04), actin filament-based movement (33.3%; 1.903e-04), contractile actin filament bundle assembly (16.7%; 2.701e-04), regulation of water loss via skin (16.7%; 5.400e-04) | 5 | 4.760E-16 | 80.89 |
| 23 | USF1 | hydrogen peroxide metabolic process (33.3%; 6.818e-05), cellular response to hydrogen peroxide (33.3%; 1.374e-04), cellular response to reactive oxygen species (33.3%; 2.572e-04), positive regulation of oxidative phosphorylation uncoupler activity (16.7%; 2.701e-04), regulation of Schwann cell migration (16.7%; 2.701e-04) | 5 | 4.760E-16 | 80.89 |
| 24 | STAT1 | regulation of cell differentiation (83.3%; 5.493e-06), regulation of developmental process (83.3%; 2.927e-05), positive regulation of cell proliferation (66.7%; 4.295e-05), regulation of Schwann cell migration (16.7%; 2.701e-04), negative regulation of cell differentiation (50.0%; 3.423e-04) | 5 | 4.760E-16 | 80.89 |
| 25 | NF-Y | antigen processing and presentation of exogenous peptide antigen via MHC class I, TAP-dependent (66.7%; 8.159e-09), antigen processing and presentation of exogenous peptide antigen via MHC class I (66.7%; 1.013e-08), antigen processing and presentation of exogenous peptide antigen (66.7%; 1.996e-08), antigen processing and presentation of exogenous antigen (66.7%; 2.309e-08), antigen processing and presentation of peptide antigen via MHC class I (66.7%; 3.209e-08) | 5 | 4.760E-16 | 80.89 |
| 26 | SMAD3 | lens fiber cell differentiation (33.3%; 1.970e-05), negative regulation of neuron projection development (33.3%; 8.011e-05), lens development in camera-type eye (33.3%; 1.627e-04), regulation of Schwann cell migration (16.7%; 2.701e-04), positive regulation of erythrocyte aggregation (16.7%; 2.701e-04) | 5 | 4.760E-16 | 80.89 |
| 27 | AP-2A | negative regulation of programmed cell death (83.3%; 4.763e-07), negative regulation of cell death (83.3%; 5.853e-07), regulation of apoptotic process (83.3%; 1.350e-05), regulation of programmed cell death (83.3%; 1.403e-05), regulation of cell death (83.3%; 1.644e-05) | 5 | 4.760E-16 | 80.89 |
| 28 | Sry | negative regulation of neuron projection development (40.0%; 5.348e-05), negative regulation of biological process (100.0%; 1.629e-04), reproductive process (80.0%; 1.653e-04), reproduction (80.0%; 1.699e-04), positive regulation of oxidative phosphorylation uncoupler activity (20.0%; 2.250e-04) | 4 | 6.960E-13 | 70.88 |
| 29 | NRF2 | response to oxidative stress (80.0%; 4.754e-07), cellular response to oxidative stress (60.0%; 2.753e-06), response to hydrogen peroxide (60.0%; 3.296e-06), response to reactive oxygen species (60.0%; 8.327e-06), negative regulation of apoptotic process (80.0%; 1.003e-05) | 4 | 6.960E-13 | 70.88 |
| 30 | ETS1 | cellular response to hydrogen peroxide (40.0%; 9.175e-05), cellular response to reactive oxygen species (40.0%; 1.719e-04), positive regulation of oxidative phosphorylation uncoupler activity (20.0%; 2.250e-04), cellular response to oxidative stress (40.0%; 4.233e-04), regulation of oxidative phosphorylation uncoupler activity (20.0%; 4.500e-04) | 4 | 6.960E-13 | 70.88 |
| 31 | c-Rel (NF-kB subunit) | response to heat (60.0%; 1.412e-06), response to temperature stimulus (60.0%; 6.985e-06), muscle cell homeostasis (40.0%; 1.116e-05), regulation of interleukin-12 production (40.0%; 7.134e-05), regulation of cytokine production (60.0%; 1.203e-04) | 4 | 6.960E-13 | 70.88 |

Gene Ontology (GO) explains the functional processes associated with built network.

zScore indicates association among the functional subnetworks of the differentially expressed proteins from 2-DE analysis.
