# Supplementary material for: Novel Targets of Sulforaphane in Primary Cardiomyocytes Identified by Proteomic Analysis
Source: PLoS One. 2013 Dec 11;8(12):e83283. doi: 10.1371/journal.pone.0083283 (PMC3859650; doi:10.1371/journal.pone.0083283)
Supplement: Supplementary file 6 [file pone.0083283.s005.doc]

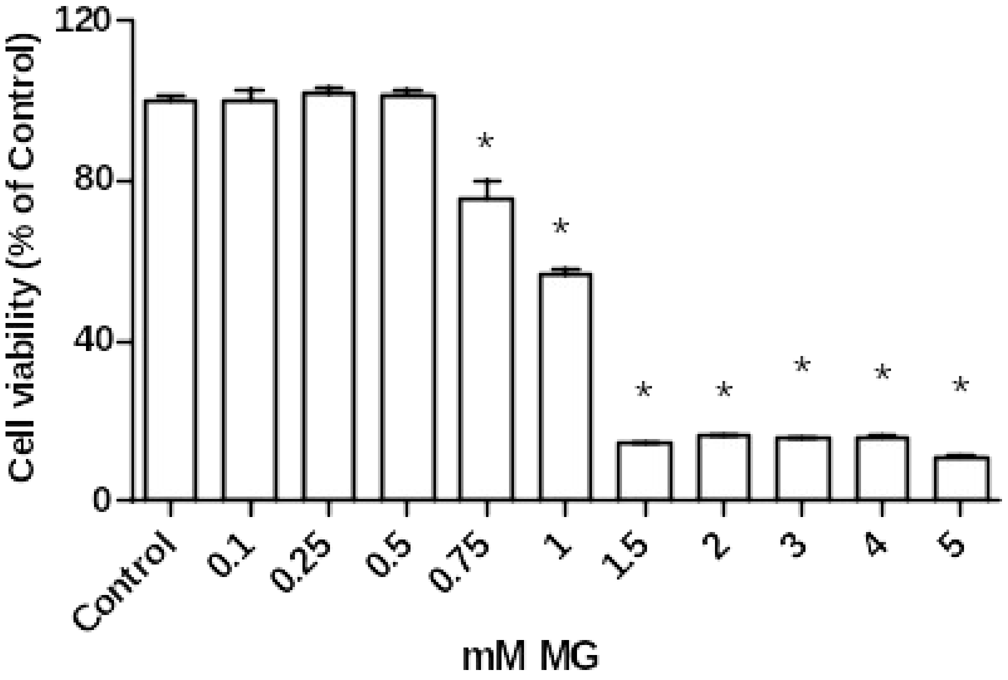

Cell viability (% of Control)
120
80
40
0
Control
0.1
0.25
0.5
0.75
1
1.5
2
3
4
5
mM MG
